# M2-like tumor-associated macrophage-secreted CCL2 facilitates gallbladder cancer stemness and metastasis

**DOI:** 10.1186/s40164-024-00550-2

**Published:** 2024-08-13

**Authors:** Weihong Chen, Mingyuan Chen, Lingju Hong, Abudukeremu Xiahenazi, Maotuan Huang, Nanhong Tang, Xinyue Yang, Feifei She, Yanling Chen

**Affiliations:** 1 Department of Hepatobiliary Surgery, Fujian Institute of Hepatobiliary Surgery, Fujian Medical University Union Hospital, Fuzhou, 350001 Fujian China; 2 Fujian Medical University Cancer Center, Fuzhou, 350108 China; 3 Key Laboratory of Ministry of Education for Gastrointestinal Cancer, Fujian Medical University, Fuzhou, 350108 China; 4 Fujian Key Laboratory of Tumor Microbiology, Department of Medical Microbiology, Fujian Medical University, Fuzhou, 350108 Fujian China

**Keywords:** M2-like tumor-associated macrophage, Gallbladder cancer, Cancer stem cells, CCL2, Epithelial-mesenchymal transformation

## Abstract

**Background:**

The predominant immune cells in solid tumors are M2-like tumor-associated macrophages (M2-like TAMs), which significantly impact the promotion of epithelial-mesenchymal transition (EMT) in tumors, enhancing stemness and facilitating tumor invasion and metastasis. However, the contribution of M2-like TAMs to tumor progression in gallbladder cancer (GBC) is partially known.

**Methods:**

Immunohistochemistry was used to evaluate the expression of M2-like TAMs and cancer stem cell (CSC) markers in 24 pairs of GBC and adjacent noncancerous tissues from patients with GBC. Subsequently, GBC cells and M2-like TAMs were co-cultured to examine the expression of CSC markers, EMT markers, and migratory behavior. Proteomics was performed on the culture supernatant of M2-like TAMs. The mechanisms underlying the induction of EMT, stemness, and metastasis in GBC by M2-like TAMs were elucidated using proteomics and transcriptomics. GBC cells were co-cultured with undifferentiated macrophages (M0) and analyzed. The therapeutic effect of gemcitabine combined with a chemokine (C-C motif) receptor 2 (CCR2) antagonist on GBC was observed in vivo.

**Results:**

The expression levels of CD68 and CD163 in M2-like TAMs and CD44 and CD133 in gallbladder cancer stem cells (GBCSCs) were increased and positively correlated in GBC tissues compared with those in neighboring noncancerous tissues. M2-like TAMs secreted a significant amount of chemotactic cytokine ligand 2 (CCL2), which activated the MEK/extracellular regulated protein kinase (ERK) pathway and enhanced SNAIL expression after binding to the receptor CCR2 on GBC cells. Activation of the ERK pathway caused nuclear translocation of ELK1, which subsequently led to increased SNAIL expression. GBCSCs mediated the recruitment and polarization of M0 into M2-like TAMs within the GBC microenvironment via CCL2 secretion. In the murine models, the combination of a CCR2 antagonist and gemcitabine efficiently inhibited the growth of subcutaneous tumors in GBC.

**Conclusions:**

The interaction between M2-like TAMs and GBC cells is mediated by the chemokine CCL2, which activates the MEK/ERK/ELK1/SNAIL pathway in GBC cells, promoting EMT, stemness, and metastasis. A combination of a CCR2 inhibitor and gemcitabine effectively suppressed the growth of subcutaneous tumors. Consequently, our study identified promising therapeutic targets and strategies for treating GBC.

**Supplementary Information:**

The online version contains supplementary material available at 10.1186/s40164-024-00550-2.

## Background

Gallbladder cancer (GBC) is the most common cancer of the biliary tract, with a dismal 5-year survival rate of only 5% owing to its aggressive nature and limited therapeutic options [1]. In most patients, metastases occur during diagnosis, further diminishing survival prospects [2]. Aggregation of immune cells within the tumor microenvironment (TME) of GBC and its impact on cancer progression [3]. Investigating the mechanisms by which the TME of GBC and its immune components affect GBC progression is crucial for improving patient outcomes.

Tumor-associated macrophages (TAMs) represent the predominant immune cell population within the TME, and they contribute to the progression of neoplastic growth [4]. These TAMs can be classified into two polarization states, namely M1-like and M2-like [5]. M2-like TAMs particularly promote tumor invasion, metastasis, epithelial-mesenchymal transition (EMT), and cancer stem cell (CSC) formation [6–8] by secreting cytokines that promote tumor growth [9, 10]. Therefore, M2-like TAMs are vital in cancer progression and exhibit intricate communication networks with tumor cells [11].Despite their significant roles, the specific contribution of M2-like TAMs to GBC progression and the underlying mechanisms require further exploration.

Aberrant EMT activation is associated with cancer progression and metastasis, which transforms tumor cells into a mesenchymal state and enhances their stemness and metastatic capability, largely driven by CSCs [12, 13]. Co-culturing tumor cells with M2-like TAMs enhanced CSC characteristics and promoted a more migratory and invasive phenotype [14]. Conversely, the depletion of M2-like TAMs inhibited the EMT process [15] and decreased the number and tumorigenicity of CSCs in solid tumors [16], which are crucial for the recruitment and polarization of M2-like TAMs [17, 18]. The complex dynamics between M2-like TAMs and tumor cells in GBC progression are partially understood.

Surgical resection is the primary curative approach for GBC, and gemcitabine-based chemotherapy is recommended for advanced-stage GBC [19]. However, these treatments are less effective against undifferentiated tumor cells and CSCs, potentially causing a high risk of tumor recurrence and metastasis [20]. The chemotactic cytokine ligan 2 (CCL2)-chemokine (C-C motif) receptor 2 (CCR2) axis activates tumor cell metastasis [21] and regulates the recruitment and polarization of immune cells, affecting cancer progression and recurrence [22]. Further research is required to elucidate the interactions between M2-like TAMs and GBC cells and the role of CCL2 in these processes.

This study focused on the role of M2-like TAMs in the progression of GBC. We found that CCL2 produced by M2-like TAMs activated the MEK/ERK signaling pathway, which enhanced GBC metastasis by regulating EMT and stemness via the ELK1/SNAIL axis. Furthermore, GBC stem cells (GBCSCs) produce CCL2, which contributes to M2-like TAM polarization and chemotaxis. These findings identified CCL2 as a pivotal mediator of M2-like TAMs and GBC cell interactions, presenting a novel target for GBC-specific therapies.

## Methods

### Clinical tissue specimens

Paraffin-embedded blocks containing GBC and adjacent non-tumor (ANT) tissues from 24 patients with GBC were prepared and collected for immunohistochemistry (IHC) to determine the expression of CD68, CD163, CD133, and CD44 (Supplementary Table 1). Additionally, freshly resected paired samples of GBC and ANT tissues from eight patients with GBC were collected to isolate CD14^+^ macrophages for subsequent cellular analyses (Supplementary Table 2). All cases were confirmed as GBC at the pathology department, and no patients underwent chemoradiotherapy before tumor resection. This study was approved by the Ethics Committee of Fujian Medical University Union Hospital (Ethical No. 2021QH019).

### Cell lines and animals

The human GBC cell lines, GBC-SD and NOZ, were acquired from the Japan Health Sciences Research Resource Bank, and the human mononuclear myeloid leukemia cell line, THP-1, was obtained from the American Type Culture Collection. All cell lines underwent STR analysis to confirm their identity and routine mycoplasma testing to ensure the absence of contamination. Male nude mice (15–20 g, 5–6 W) from Guangdong Pharmachem Biotechnology Co., Ltd. were housed in the Fujian Medical University Experimental Animal Center. The mice were subjected to a 12-h light-dark cycle for feeding. The Fujian Medical University Ethics Committee approved this study (Ethics No. FJMU IACUC 2021 − 0383).

### Magnetic activated cell sorting for macrophages

The samples were processed using eye scissors to obtain the dimensions of 1 × 1 × 1 mm. The digestion process was conducted at 37 °C using a digestion solution according to the manufacturer’s instructions (130-095-929, Miltenyi). Ficoll^®^ solution (26873-85-8, MERCK) was used to separate the suspension. Macrophages were isolated from lymphocytes through treatment with the EasySep™ Human CD14 Positive Selection Kit II (17858 100–0694 17858RF, Stemcells).

### Cell culture and induction

GBC-SD, NOZ, and THP-1 cells were cultured in Dulbeco’s Modified Eagles’s Medium (12100046, Gibco) or RPMI 1640 medium (11875101, Gibco) supplemented with 10% fetal bovine serum (FBS) (10099141 C, Gibco). The cells were incubated in a humidified atmosphere at 37 °C and 5% CO_2_. When the GBC cell density reached 90%, the medium was discarded, and trypsin was added (25200072, Gibco) for a 3-min digestion. Subsequently, the cells were centrifuged at 800 × *g* for 5 min, and a complete medium was added for passaging. After a 48-h culturing of THP-1 cells, the cells were centrifuged at 800 × *g* for 5 min, and the medium was added to the passenger culture. Next, the THP-1 cells were revulsed into undifferentiated macrophages (M0) and M2-like TAMs through the application of 100 ng/mL PMA (16561-29-8, MERCK), 20 ng/mL IL-4 (200-04, PeproTech), and 20 ng/mL IL-13 (200 − 13, PeproTech) [23]. RPMI 1640 medium supplemented with 2% FBS was introduced to M0 and M2-like TAMs. The resulting supernatant was obtained after 24 h through centrifugation at 2,000 × *g* for 5 min and stored at -80 °C if not immediately used. Depending on the specific groups, GBC cells were subjected to treatment with 20 µM U0126 [24] (19–147, MERCK), 20 µM RS504393 [25] (300816-15-3, MERCK), and 150 ng/mL CCL2 [26] (300 − 36, PeproTech) for 48 h.

### IHC

Tissue samples were prepared by cutting them into blocks of approximately 5 × 5 × 3 mm and embedding them in wax molds. Subsequently, the blocks were sliced using a microtome after being pre-cooled for 10–15 min. The paraffin sections were deparaffinized, and endogenous peroxidase was blocked. Sections were incubated with a drop of 3% bovine serum albumin (BSA) (9048-46-8, MERCK) and titrated with the primary antibody (Supplementary Table 3). The sections were carefully dried, followed by the dropwise addition of secondary antibodies (as listed in Supplementary Table 3) specific to the species corresponding to the designated circles. Color development was performed, the nuclei were stained, and the sections were dehydrated and sealed. At least five fields of view from each section were carefully examined under a microscope at 400× magnification. Immunoreactivity score (IRS) = staining intensity (SI) × percentage of positive cells (PP). The SI was scored as 0 = negative, 1 = weak, 2 = moderate, and 3 = strong. PP was defined as 0 = 0%; 1 = 0–25%; 2 = 25–50%; 3 = 50–75%; 4 = 75–100%. All patients were divided into two groups based on the median expression score (high expression group: > median score; low expression group: ≤ median score) [27, 28].

### Sphere-forming assay

GBC cells (2 × 10^3^ ) were collected and cultured in low-adhesion culture plates (3471; Corning). The cells were cultured in serum-free DMEM/F-20 (21331020, Merck) supplemented with 12 ng/mL FGF (100-18B, PeproTech), 20 ng/mL EGF (100 − 47, PeproTech), and 20 ng/mL IGF (100 − 11, PeproTech). The incubation was performed at 37 °C and 5% CO_2_ in a humidified environment for 7 days according to the manufacturer’s guidelines. The resulting cell cultures were examined under an inverted microscope. Three random fields of view were selected and analyzed to determine the sphere formation efficiency (SFE).

### Flow cytometry and fluorescence-activated cell sorting

For surface staining, the cells were directly incubated with antibodies (Supplementary Table 3). For intracellular staining, cells were first subjected to surface staining, followed by fixation and permeabilization (554722, BD Biosciences). Subsequently, the cells were washed twice with 1× BD Perm/Wash™ Buffer (554723, BD) and treated with antibodys (Supplementary Table 3). All samples were washed twice with phosphate-buffered saline (PBS) containing 2% FBS. Sorting and analysis were performed using a fluorescence-activated cell sorting or Celesta flow cytometer, and the FlowJo software was used for data analysis.

### Western blotting

Cells were lysed using RIPA lysis buffer (V900854, Merck) and PMSF (329-98-6, Merck) to extract total protein. Nuclear and non-nuclear proteins were extracted separately according to the manufacturer’s instructions (R0050, Solarbio). The protein concentration was determined using the BCA assay (71285-M, Merck) following the manufacturer’s instructions. Total proteins were separated through Sodium Deodocyl Sulfate Polyacrylamide Gel Electrophoresis and transferred onto a Polyvinylidene Fluoride (PVDF) membrane (IEVH00005, Millipore). The PVDF membranes were blocked with BSA for 30 min and incubated with primary (Supplementary Table 3) and secondary (Supplementary Table 3) antibodies. The resulting color development was achieved using an ECL-Luminescence solution (P0018S, Beyotime), and the bands were detected using the Bio-Rad ChemiDoc XRS + system. The net gray value of these bands was determined using the ImageJ software.

### Cytokine array analysis

Culture supernatants of M0- and M2-like TAMs were collected individually. The array membranes were processed following the manufacturer’s instructions (ab 133998, Abcam) and subjected to detection using a detection buffer on a Bio-Rad ChemiDoc XRS + system. The net gray values of the membranes were quantified using the ImageJ software.

### Proteomics and RNA-seq

The LC-MS/MS analysis was performed using a Q-Exactive mass spectrometer. The mass spectrometer was operated in the positive ion mode. MS data were acquired using a data-dependent top20 method, dynamically choosing the most abundant precursor ions from the survey scan (300–1800 m/z) for HCD fragmentation. The automatic gain control target was set to 1e6, and the maximum injection time was set to 50 ms. The dynamic exclusion duration was 30 s. Survey scans were acquired at a resolution of 60,000 at m/z 200, the resolution for the HCD spectra was set to 15,000 at m/z 200, and the isolation width was 1.5 m/z. The normalized collision energy was 30 eV, and the underfill ratio, which specifies the minimum percentage of the target value likely to be reached at the maximum fill time, was defined as 0.1%. RNA-seq was performed by the Wuhan Metware Biotechnology Co.

### Enzyme-linked immunosorbent assay (ELISA)

A volume of 200 µL of standard and sample was added into each well, followed by washing and incubation with 200 µL of CCL2 conjugate (DCP00, RD) for 1 h. Subsequently, 200 µL of substrate solution was added in a light-free environment, and the optical density was assessed at wavelengths of 450 nm and calibration wavelengths of 540–570 nm.

### RNA preparation and quantitative reverse-transcription PCR

Total RNA was extracted using TRIzol reagent (T9424, Merck) following the manufacturer’s instructions. For qPCR, cDNA was synthesized by adding TransScript^®^ II One-Step gDNA Removal and cDNA Synthesis SuperMix (AH311-02, TransGen) to the PCR tube. The amplification process involved a holding stage at 95 ℃ for 10 s, followed by a cycling stage at 95 ℃ for 10 s, 60 ℃ for 20 s, and 72 ℃ for 36 s, repeated in 40 cycles. Relative expression was determined using the 2^−ΔΔCt^ method. The lysis curve was plotted using the default instrument program. The primer sequences used in this study are listed in Supplementary Table 4.

### Mouse studies and bioluminescence imaging

NOZ cells, which were in the logarithmic growth phase and had successfully induced M2-like TAMs, were resuspended in PBS. The concentration of the single-cell suspension was adjusted to 5 × 10^6^ cells/mL, and the M2 co-culture group comprised GBC cells and M2-like TAMs at a ratio of 1:1. The subcutaneous xenograft or lung metastasis model was established by injecting 200 µL of tumor cells suspension into the right axilla or tail vein, respectively. After 10 days, peritumoral and intraperitoneal injections of 800 µg/kg CCL2, 20 mg/kg RS504393, and 100 mg/kg gemcitabine (122111-03-9, Merck) were administered every 3 days depending on the group. To evaluate tumor growth, the subcutaneous tumor model involved seven administrations before sacrifice, and the lung metastasis model involved 14 administrations. The volume and weight of the tumor tissues were measured. In addition, tumors and lung tissues were examined using hematoxylin and eosin staining and IHC. The luciferase-tagged lentivirus used in this study was constructed, synthesized, and characterized by Shanghai Genechem Co. Ltd. For imaging purposes, a 150 mg/kg dose of D-fluorescein potassium salt (115144-35-9, Sigma) dissolved in PBS at 30 mg/mL was administered via intraperitoneal injection and allowed to circulate for 10 min. Imaging was performed using an Image Visualization And Infrared Spectroscopy Lumina Series III. The survival rate of experimental mice was assessed following the Wistar IACUC guidelines for sedation, analgesia, anesthesia, and euthanasia.

### Immunofluorescence

GBC cells were cultured on slides and immobilized using a fixative (P0098; Beyotime). Subsequently, the sections were exposed to immunostaining permeabilization (P0095, Beyotime) and blocking (P0260, Beyotime) solutions, followed by incubation with labeled primary antibodies (Supplementary Table 3). Fluorescent secondary antibodies (Supplementary Table 3) were used to label the pairs of sections. DAPI (C1002, Beyotime) was used to re-stain the nuclei. The samples were scanned with a confocal laser microscope, with emission wavelengths set at 488 and 555 nm and excitation wavelengths set at 530 and 580 nm. For each section, five fields were randomly analyzed.

### Double luciferase reporter gene assay

Cells were lysed according to the manufacturer’s instructions (PM040; Promega). Subsequently, 1 × PLB lysate was introduced for 15 min and transferred to an opaque white 96-well plate, and LARII was added. The amount of luciferase-excited substrate release from the firefly was determined. Next, Stop&Glo Reagent was added, and the amount of the sea cucumber luciferase-excited substrate release was determined.

### Chromatin immunoprecipitation (CHIP) assays

Chromatin was cross-linked, and cells were collected and lysed using Cell Lysis Buffer (17-10085, Merck) according to the manufacturer’s instructions. The cross-linked DNA fragments were sonicated to achieve a length range of 200–1,000 bp. ELK-1 and IgG antibodies were introduced (Supplementary Table 3). The DNA was purified through uncrossing. Finally, the purified DNA was subjected to qPCR using specific primers (Supplementary Table 4) for detection.

### Plasmid transfection

The recombinant SNAIL promoter-reporter gene vector, recombinant reporter gene vector for each truncated fragment, recombinant SNAIL binding site mutant vector, eukaryotic ELK-1 expression plasmids, sh-ELK-1, and sh-SNAIL were synthesized and characterized by Shanghai GeneChem Co., Ltd. (Supplementary Table 5). GBC cells were transformed using Invitrogen™ Lipofectamine™ 3000 Transfection Reagent (L3000075, Thermo) following the manufacturer’s instructions. All sequences were verified using DNA sequencing.

### Migration assay

GBC cells were co-cultured with M0- or M2-like TAMs. The lower chamber of a Transwell (3422, Corning) was filled with 400 µL of various media, while the upper chamber was filled with cells and incubated in a humidified atmosphere with 5% CO_2_ at 37 ℃ for 24 h. The Transwell was removed and treated with a fixative (G1101, Servicebio) for 30 min. Subsequently, the Transwell was stained with 500 µL of 0.1% crystal violet (G1014, Servicebio) for 20 min. The number of cells in five fields of view was assessed using a microscope, and cell migration was compared between the different experimental groups. After a 48-h incubation of GBC cells with different drugs based on their respective groups, 1 × 10^6^ cells were seeded onto a plate, and a wound was created. The wounds were examined under a microscope at 0 and 24 h, and the wound healing rate was calculated.

### Statistical analysis

Statistical graphs were created using GraphPad Prism 9.0. Measurements were presented as mean ± standard deviation (SD). Data were analyzed using Student’s *t-test* to compare means between two groups with normal distribution, one-way analysis of variance to compare means between several groups, and the chi-square test for correlation analysis. Statistical significance was set at a significance level of *P* < 0.05.

### Flow chart of the study

A flowchart was designed to offer a clear and concise overview of the study’s methodology, highlighting the sequence of events and experimental approaches used (Supplementary Fig. 1).

## Results

### Increased expression of CSC and M2-like TAM markers in GBC correlates with metastasis

**Fig. 1:**
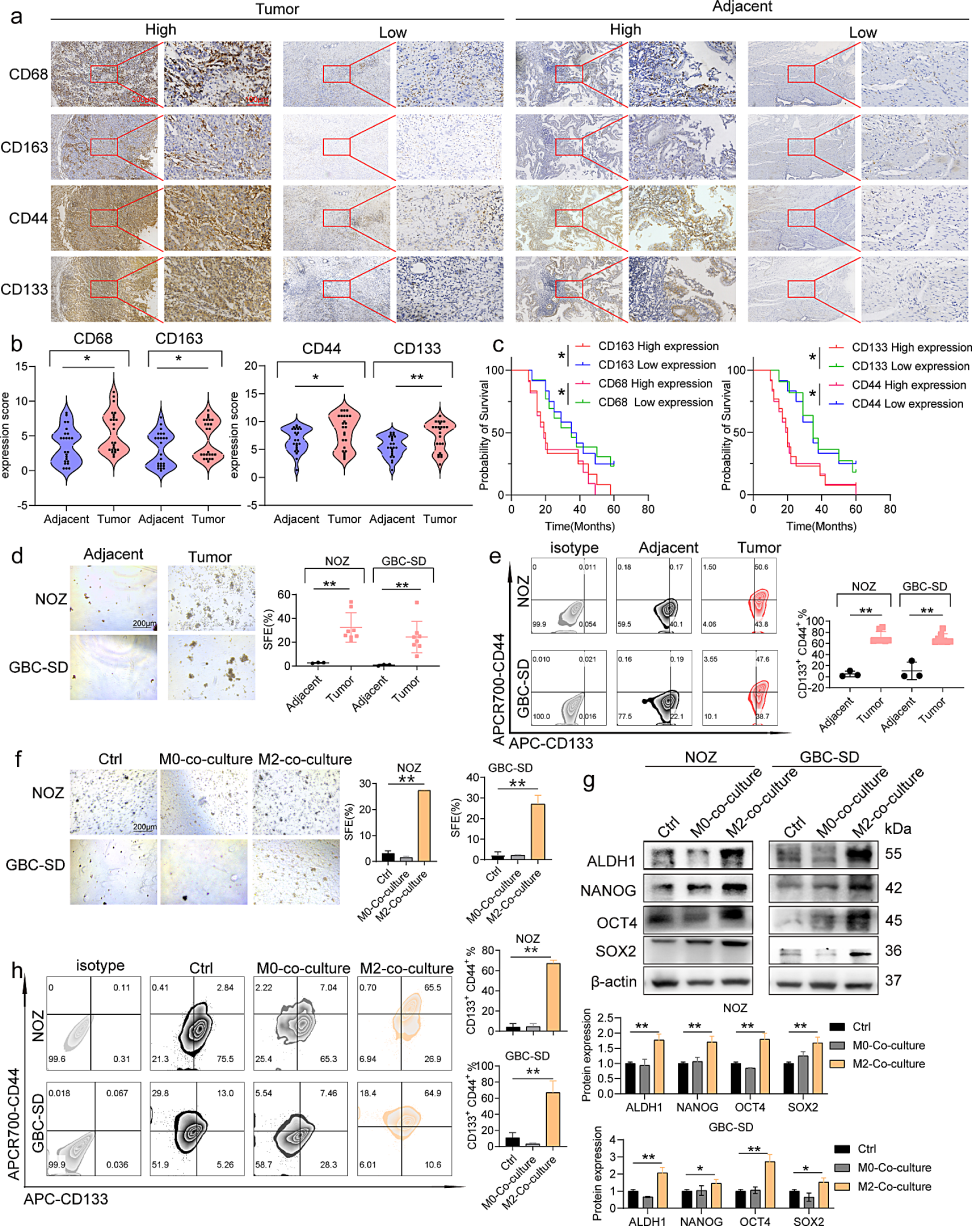
Increased expression of CSCs and M2-like TAM markers in GBC is associated with metastasis (**a**) Representative immunohistochemical images of CD163, CD68, CD44, and CD133 in GBC and adjacent non-cancerous tissues in 24 patients with GBC (*n* = 24). (**b**) Expression of CD163, CD68, CD44, and CD133 in GBC and adjacent non-cancerous tissues from 24 patients with GBC (*n* = 24). (**c**) Expression of CD163, CD68, CD44, and CD133 and patient survival rates (*n* = 24). (**d**) SFE of NOZ and GBC-SD cells co-cultured with CD14^+^ TAMs obtained from fresh tissues of patients with GBC (*n* = 8). (**e**) Flow cytometry of the proportion of CD133^+^ CD44^+^ in NOZ and GBC-SD cells co-cultured with macrophages obtained from fresh tissues of patients with GBC (*n* = 8). (**f**) SFE in GBC cells co-cultured with M2-like TAMs (*n* = 3). (**g**) Western blotting of ALDH1, NANOG, SOX2, and OCT4 expression in GBC cells (*n* = 3). (**h**) Detection of the proportion of CD133^+^ CD44^+^ in GBC cells co-cultured with M2-like TAMs (*n* = 3). Data are presented as mean ± standard deviation (SD). GBC: gallbladder cancer, M2:M2-like tumor-associated macrophage, SFE: Sphere Formation Efficiency. Statistical significance was assessed using the Student’s *t-test* (b, d, e, f, g, h) and log-rank test (c). *P < 0.05, **P < 0.01

High expression of M2-like TAM [29] and CSC [30] markers is associated with adverse clinical outcomes in various solid tumors. To assess their expression and association with GBC prognosis, we observed significantly increased levels of CD163, CD68, CD44, and CD133 in GBC tissues compared with those in adjacent non-cancerous tissues (Fig. 1a). Correlation analysis revealed a positive correlation among CD163, CD68, CD44, and CD133 expression in GBC tissues (Supplementary Fig. 2a). In addition, CD163 and CD68 expression in GBC tissues was correlated with TNM stage and nerve metastasis (Supplementary Table 6). However, no significant association was observed between CD163 and CD68 expression and age or sex. Similarly, CD44 and CD133 expression in GBC tissues were correlated with tumor size and lymphatic metastasis but not with age or sex (Supplementary Table 7). Notably, patients with high CD163, CD68, CD44, and CD133 expression levels had lower survival rates than those with low expression levels (Fig. 1b). These indicate a significant upregulation of CD163, CD68, CD44, and CD133 in GBC tissues, which is positively associated with metastasis in the affected patients. This suggests a possible relationship between CSCs and M2-like TAMs in GBC.

Owing to the positive correlation among CD163, CD68, CD44, and CD133 expression in GBC tissues, we isolated CD14^+^ TAMs from patients with GBC [31]. Subsequently, CD14^+^ TAMs were co-cultured with GBC cells (Supplementary Fig. 2c). CD14 is significant in identifying M2-like TAMs [32]. Notably, our results suggest that CD14^+^ TAMs significantly enhanced tumor SFE and the proportion of CD133^+^ CD44^+^ cells in GBC cells (Fig. 1d and e). The macrophages in adjacent non-cancerous tissues resulted in decreased SFE and proportion of CD133^+^ CD44^+^ GBC cells (Fig. 1d and e; Supplementary Fig. 2b provides the gate-drawing logic for this analysis). To further investigate this phenomenon, GBC cells were co-cultured with M2-like TAMs induced by THP-1 cells in vitro. The tumorsphere assay showed that the presence of M2-like TAMs increased the tumor SFE in GBC cells (Fig. 1f). Furthermore, co-culturing of GBC cells with M2-like TAMs increased the proportion of CD133^+^ CD44^+^ GBCSCs in the GBC cell population (Fig. 1h). The upregulation of ALDH1, NANOG, SOX2, and OCT4 was observed in GBC cells in response to M2-like TAMs (Fig. 1g). These suggest that M2-like TAMs can improve the stemness of GBC cells.

The initiation of EMT is closely associated with the conversion of tumor cells to CSCs [33]. In a co-culture environment with GBC cells, M2-like TAMs, and undifferentiated M_0_ cells, there was significant upregulation of the mesenchymal-associated proteins N-Cadherin and Vimentin, accompanied by a downregulation of the epithelial-associated protein E-cadherin in GBC cells (Supplementary Fig. 2d). In addition, the expression levels of CD163, CD68, CD44, and CD133 in GBC tissues were significantly correlated with tumor metastasis. GBC cells showed incremental migration toward the lower compartment of the co-culture system and increased wound healing rate (Supplementary Fig. 2e–g). These indicate that M2-like TAMs play a role in promoting the EMT and migration of GBC cells.

#### M2-like TAMs drive GBC cell EMT, stemness, and migration by secreting CCL2

**Fig. 2:**
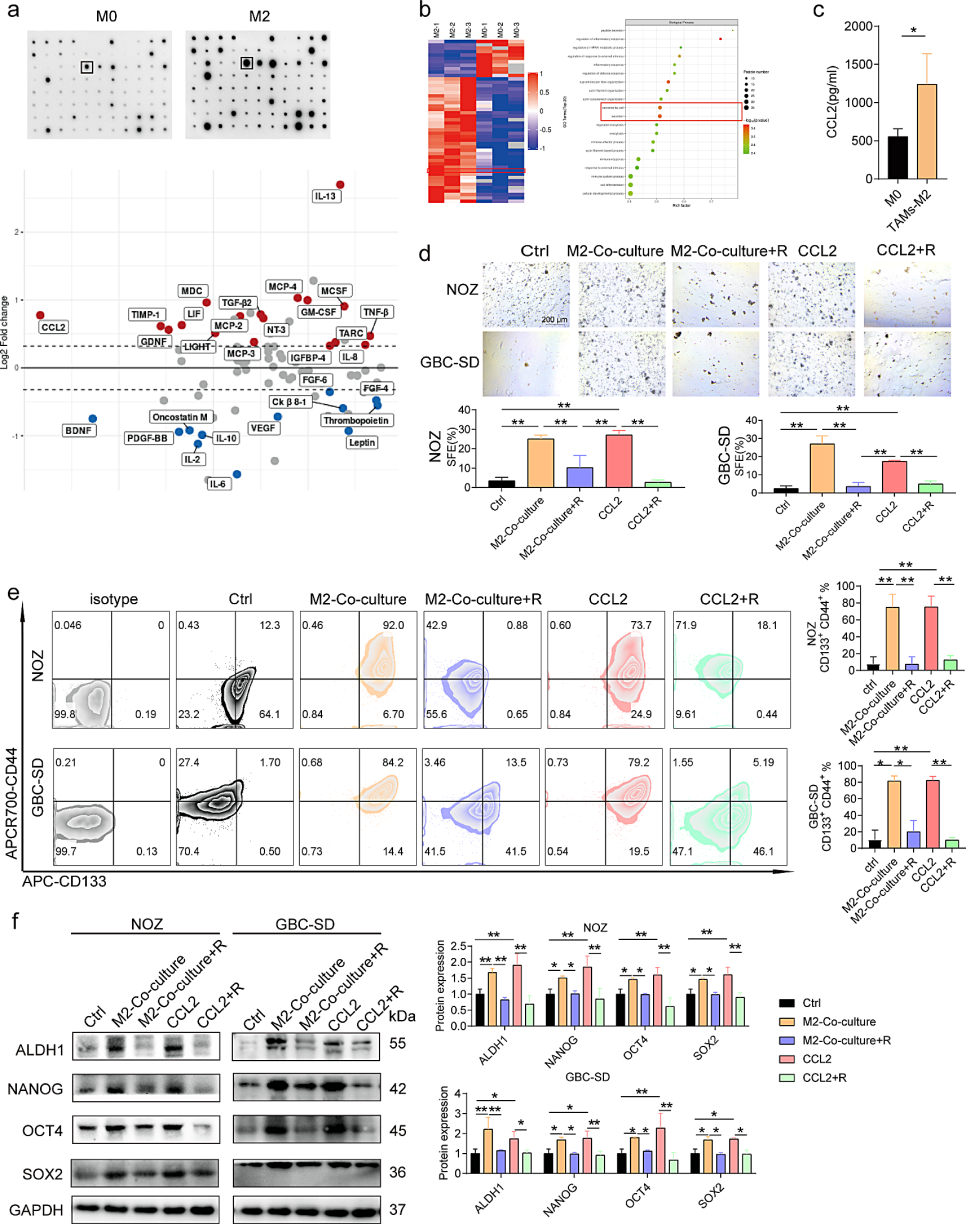
M2-like TAMs drive GBC cell EMT, stemness, and migration by secreting CCL2. (**a**) Cytokine array analysis was used to detect cytokines in the supernatants of M0 and M2-like TAMs (*n* = 3). (**b**) Proteomics was used to identify cytokines in the supernatants of M0 and M2-like TAMs (*n* = 3). (**c**) ELISA was used to determine CCL2 expression in M0 and M2-like TAMs (*n* = 3). (**d**) SFE in GBC cells was determined using tumor sphere formation assay (*n* = 3). (**e**) Flow cytometry was used to measure the proportion of CD133^+^ CD44^+^ GBC cells (*n* = 3). (**f**) Western blotting was performed to determine the expression of ALDH1, NANOG, SOX2, and OCT4 in GBC cells (*n* = 3). Data are presented as mean ± SD. ELISA: Enzyme-linked immunosorbent assay, GBC: gallbladder cancer, M2:M2-like tumor-associated macrophage, R: RS504393, SFE: Sphere Formation Efficiency. Statistical significance was assessed using the Student’s *t-test* (a–f). *P < 0.05, **P < 0.01

M2-like TAMs reportedly promote EMT, stemness, and migration of GBC cells. Therefore, we investigated the mechanisms underlying the contribution of M2-like TAMs in GBC progression. First, cytokine array analysis was performed, which confirmed a significant increase in CCL2 expression in M2-like TAMs (Fig. 2a). Subsequently, proteomic analysis was performed on the supernatants of M0- and M2-like TAMs, which revealed a significant increase in CCL2 levels in the latter (Fig. 2b). This finding was further supported by ELISA and qPCR validation, which showed higher levels of CCL2 expression in M2-like TAMs (Fig. 2c and Supplementary Fig. 3a). Moreover, we analyzed the significant upregulation expression of CCL2 in freshly obtained tissue macrophages and serum samples from patients with GBC (Supplementary Fig. 3b and c).

The significant expression levels of the cytokine CCL2 in M2-like TAMs suggest that it plays a regulatory role in the EMT, stemness, and migration of GBC cells. To examine the function of CCL2, we administered RS504393, an antagonist of the CCL2 receptor, CCR2. Our results suggest that GBC cells showed upregulated N-cadherin and vimentin and downregulated E-cadherin after exposure to M2-like TAMs and CCL2 (Supplementary Fig. 3d). The effects of M2-like TAMs were attenuated by RS504393 treatment (Supplementary Fig. 3d). SFE was enhanced by M2-like TAMs and CCL2; however, it was decreased when RS504393 was used in GBC cells (Fig. 2d). Co-culturing M2-like TAMs with GBC cells caused a significant increase in the proportion of CD133^+^ CD44^+^ GBC cells, whereas the administration of RS504393 resulted in a decrease (Fig. 2e). Furthermore, M2-like TAMs and CCL2 were significant in increased expression of ALDH1, NANOG, SOX2, and OCT4 in GBC cells, whereas the effect of M2-like TAMs was reversed by RS504393 (Fig. 2f). Following these findings, the migration assays showed that the presence of M2-like TAMs and CCL2 enhanced migration, whereas the administration of RS504393 had a suppressive effect on GBC cell migration (Supplementary Fig. 3e and f). Thus, CCL2 plays a significant role in facilitating the induction of EMT, stemness, and the migration of GBC cells via M2-like TAMs.

#### M2-like TAMs promote GBC EMT, stemness, and metastasis in vivo by secreting CCL2

**Fig. 3:**
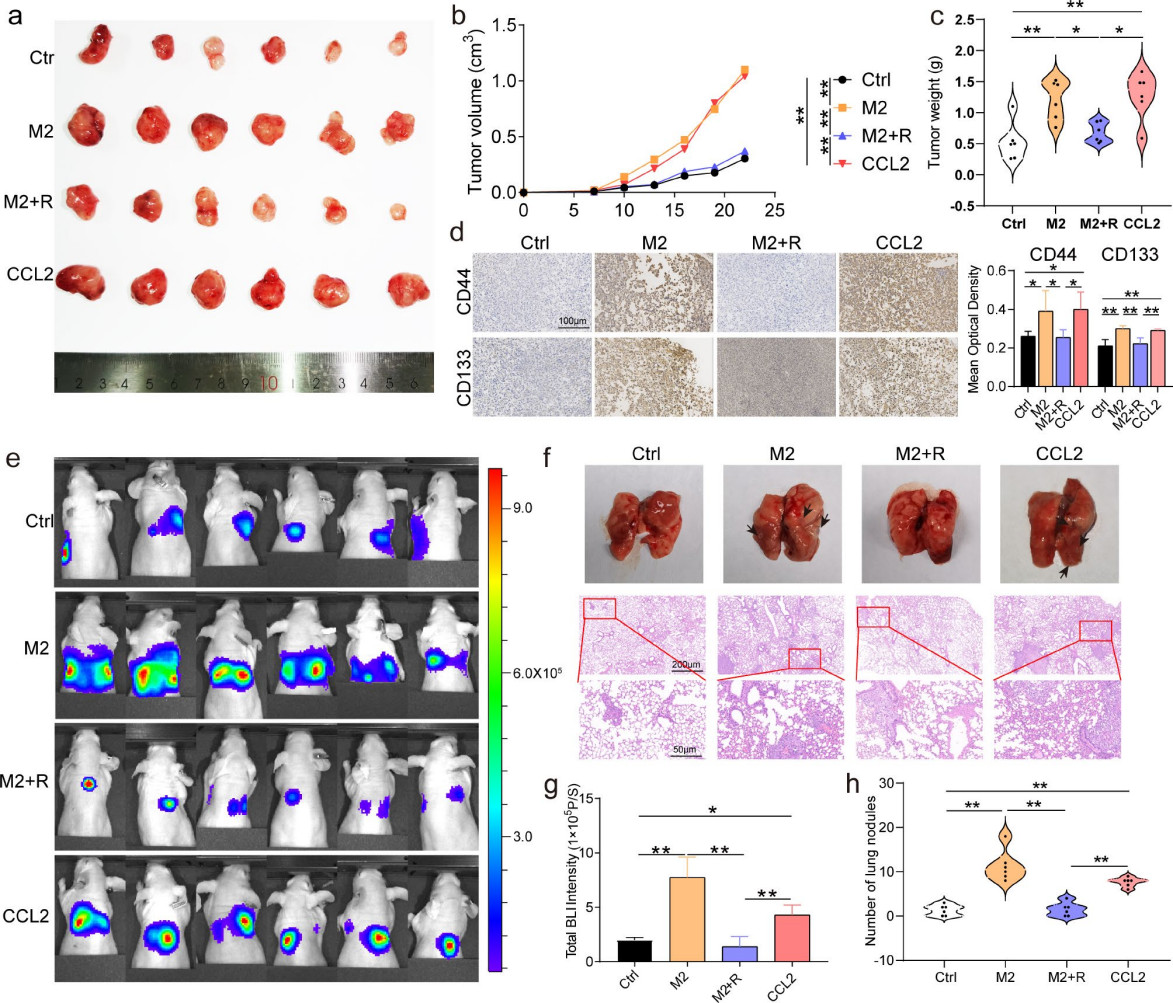
M2-like TAMs promote GBC EMT, stemness, and metastasis in vivo through the secretion of CCL2. (**a**) Images of subcutaneous tumors (*n* = 6). (**b**) Weight of subcutaneous tumors (*n* = 6). (**c**) Growth curve of subcutaneous tumors (*n* = 6). (**d**) Immunohistochemistry of CD44 and CD133 expression in subcutaneous tumors (*n* = 6). (**e**) Images of BLI in the lung metastasis model (*n* = 6). (**f**) Images of lung metastasis tumor (*n* = 6). (**g**) Total BLI intensity of lung metastases (*n* = 6). (**h**) Number of lung metastases tumor (*n* = 6). Data are presented as mean ± SD. BLI: bioluminescence, GBC: gallbladder cancer, M2:M2-like tumor-associated macrophage, R: RS504393, BLI: Bioluminescence image. Statistical significance was assessed using Student’s *t-test* (b, c, d, g, h). *P < 0.05, **P < 0.01

To further confirm the promotion of GBC progression by M2-like TAMs through CCL2 secretion in vivo, we established subcutaneous transplant tumor and lung metastasis models to observe the growth and metastasis of GBC in vivo (NOZ-luciferase showed no difference in migration and proliferation compared with NOZ, Supplementary Fig. 4a and b). Simultaneous injection of M2-like TAMs and GBC cells caused accelerated tumor growth, and this tumor-promoting effect of M2-like TAMs was counteracted by the administration of RS504393 (Fig. 3a–c). Similarly, CCL2 administration increased tumor growth (Fig. 3a–c). Consistent with the in vivo observations, the introduction of M2-like TAMs caused the upregulation of N-cadherin, vimentin, CD44, and CD133, whereas the expression of E-cadherin was downregulated in tumors (Fig. 3d and Supplementary Fig. 4c). However, this effect was attenuated by RS504393 treatment (Fig. 3d and Supplementary Fig. 4c). Furthermore, CCL2 increased the expression of N-cadherin, vimentin, CD44, and CD133 and decreased those of E-cadherin (Fig. 3d and Supplementary Fig. 4c). In the lung metastasis model, the injection of M2-like TAMs and RS504393 caused lower total bioluminescence (BLI) intensity than that in lung tumors injected with M2-like TAMs alone (Fig. 3e and f). Furthermore, the presence of CCL2 caused an increase in the total BLI intensity of the tumors (Fig. 3e and f). Co-administration of M2-like TAMs increased the incidence of lung neoplasia, and CCL2 also increased the number of tumors (Fig. 3g). However, the M2-like TAMs-induced increase in lung tumor number was counteracted by the administration of RS504393 (Fig. 3g). Therefore, the in vivo experiments emphasizes that M2-like TAMs facilitate GBC EMT, stemness, and metastasis through the release of CCL2.

#### M2-like TAMs promote GBC EMT, stemness, and migration via CCL2/MEK/ERK

**Fig. 4:**
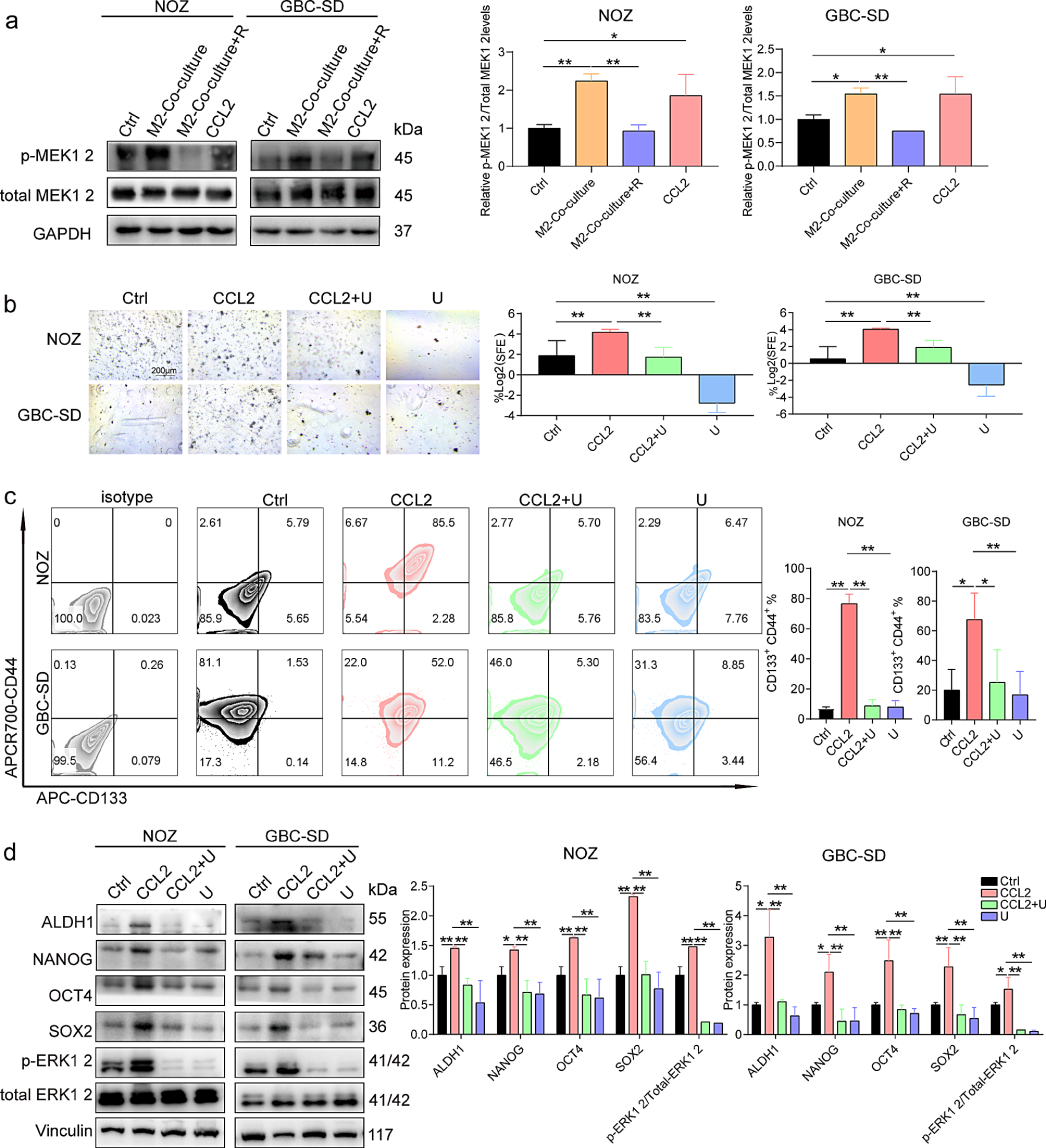
M2-like TAMs promote GBC EMT, stemness, and migration via CCL2/MEK/ERK. (**a**) Western blotting was used to identify the expression of p-MEK and total-MEK in GBC cells (*n* = 3). (**b**) SFE of GBC cells (*n* = 3). (**c**) Flow cytometry was used to determine the proportion of CD133^+^ CD44^+^ in GBC cells (*n* = 3). (**d**) Western blotting was performed to examine the expression of ALDH1, NANOG, SOX2, and OCT4 in GBC cells (*n* = 3). Data are presented as mean ± SD. GBC: gallbladder cancer, M2:M2-like tumor-associated macrophage, R: RS504393, U: U0126, SFE: Sphere Formation Efficiency. Statistical significance was assessed using the Student’s *t-test* (a–d). *P < 0.05, **P < 0.01

RNA-seq was performed on GBC cells co-cultured with M2-like TAMs to investigate the mechanism by which CCL2 promotes GBC EMT, stemness, and metastasis. Kyoto Encyclopedia of Genes and Genome analysis showed enrichment of the differential gene associated with the MEK pathway (Supplementary Fig. 5a). Western blotting was used to validate the expression of proteins in the AKT [34], JNK [35], WNT β-catenin [36], SHH [37], and MEK/ERK [38] pathways, which are influenced by CCL2. The phosphorylated MEK showed increased reactivity towards M2-like TAMs or CCL2, and this effect was counteracted by RS504393 (Fig. 4a). Conversely, the expression levels of proteins in other signaling pathways showed negligible changes (Supplementary Fig. 5b). Consequently, the enhancement of GBC progression by M2-like TAMs can be attributed to the CCL2/MEK/ERK signaling cascade.

U0126, a MEK inhibitor, was used to validate the involvement of CCL2 in GBC progression through the MEK/ERK signaling pathway. In GBC cells, CCL2 upregulated the expression of N-cadherin and vimentin and downregulated that of E-cadherin (Supplementary Fig. 6a). Administration of U0126 inhibited EMT (Supplementary Fig. 6a). Additionally, CCL2 favored GBC cell stemness, as evidenced by increased SFE and the proportion of CD133^+^ CD44^+^ cells in GBC cells (Fig. 4b and c). Moreover, CCL2 facilitated the expression of ALDH1, NANOG, SOX2, and OCT4 (Fig. 4b–d). In contrast, administration of U0126 caused a reduction in SFE, the proportion of CD133^+^ CD44^+^ GBC cells, and the expression of ALDH1, NANOG, SOX2, and OCT4 in GBC cells (Fig. 4b and c). Furthermore, migration experiments revealed that CCL2 facilitated the migration of GBC cells into the lower compartment and promoted wound healing (Supplementary Fig. 6b–d). However, the migratory capacity of GBC cells was attenuated in the presence of U0126 (Supplementary Fig. 6b–d). These results suggest that CCL2 exerts its effect on GBC cells through the activation of the MEK/ERK signaling pathway, promoting EMT, stemness, and migration in GBC cells.

#### CCL2 activates the MEK/ERK/ELK1/SNAIL pathway in GBC cells

Previous studies have shown that EMT transcription factors (EMT-TFs) play a role in regulating EMT and are closely linked to functioning in CSCs through the modulation of gene expression, including CD44, CD133, and SOX2 [39, 40]. The upregulation of EMT-TFs can reprogram cancer cells from a differentiated to stem cell state [39, 40]. We observed an increased SNAIL expression in GBC cells in response to ERK activation, and this ERK-induced SNAIL promotion was inhibited by U0126. However, ERK and U0126 activation had no significant effects on other EMT-TFs (Supplementary Fig. 7a and b). Activation of MEK/ERK signaling leads to the stimulation of SNAIL expression by CCL2; however, SNAIL is not directly regulated by ERK [41]. To identify the regulatory factors of SNAIL, we used PROMO [42] and compared the results with those of the downstream factors of MEK/ERK that were predicted in a previous study (Supplementary Material 2) [41]. Consequently, we identified 10 transcriptional regulators of SNAIL via the CCL2/MEK/ERK pathway (Supplementary Fig. 7c). Among these regulators, ERK can phosphorylate ELK1 through direct interactions with the two proteins [43]. Therefore, we hypothesized that the CCL2/MEK/ERK pathway enhances SNAIL expression in GBC cells by facilitating ELK1 phosphorylation and subsequent nuclear translocation.

**Fig. 5:**
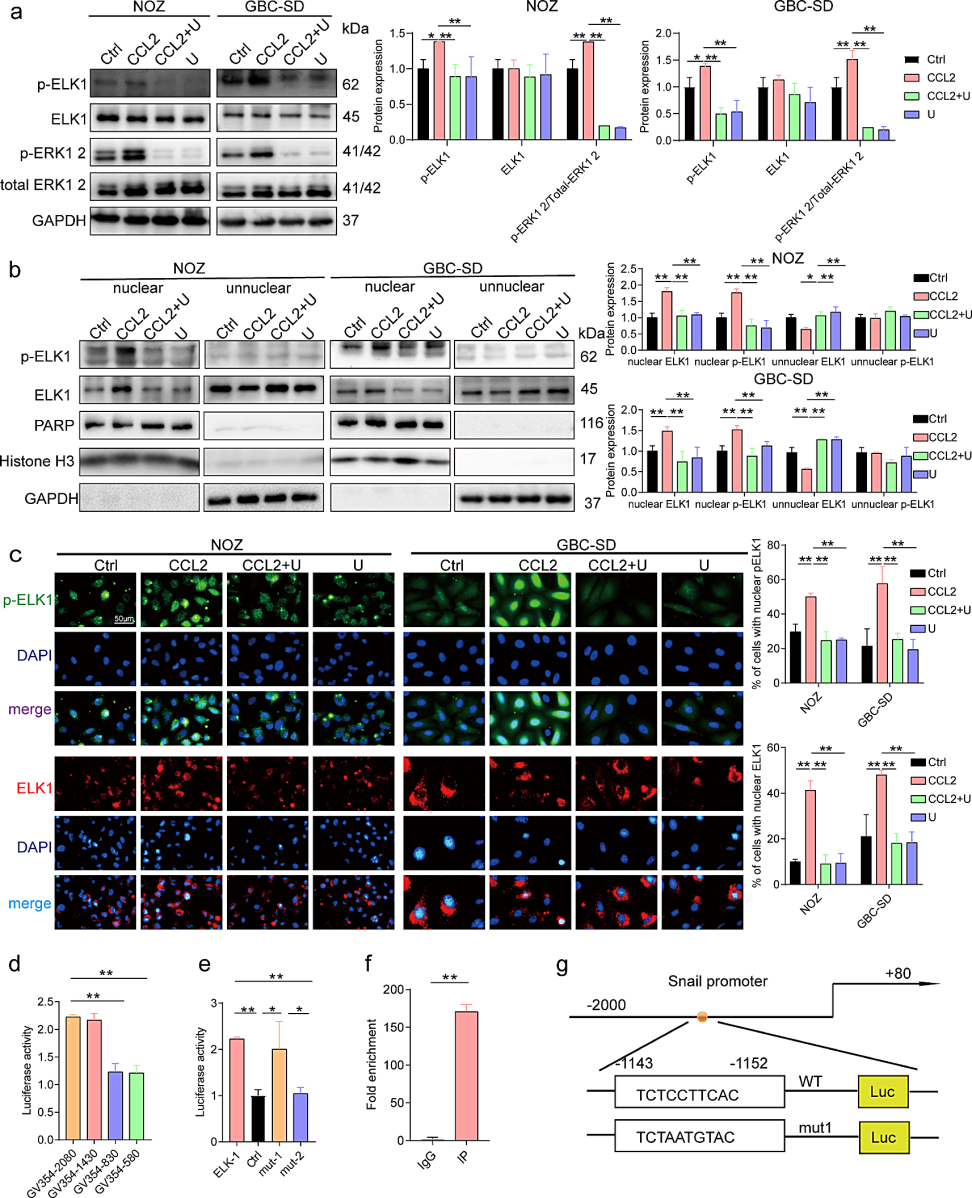
CCL2 activates the MEK/ERK/ELK1/SNAIL signaling pathway in GBC cells. (**a**) Western blotting for ELK1, p-ELK1, ERK, and p-ERK expression in GBC cells (*n* = 3). (**b**) Western blotting showed increased intranuclear expression of ELK1 and p-ELK1 in GBC cells (*n* = 3). (**c**) Immunofluorescence staining was used to determine the expression of ELK1 and p-ELK1 in GBC cells (*n* = 3). (**d**) Double luciferase reporter gene assay for the fluorescence intensity of truncated fragments (*n* = 3). (**e**) Double luciferase reporter gene assay was used to measure the fluorescence intensity of mutant fragments (*n* = 3). (**f**) CHIP validated the binding sites (*n* = 3). (**g**) The diagram of the ELK1 binding site on the SNAIL promoter. Data are presented as mean ± SD. CHIP: Chromatin immunoprecipitation, GBC: gallbladder cancer, U: U0126. Statistical significance was assessed using the Student’s *t-test* (a–f). *P < 0.05, **P < 0.01

Our results support the hypothesis that ERK activation in GBC cells causes increased p-ELK1 expression (Fig. 5a). Furthermore, our observations showed that ERK activation facilitated nuclear translocation of ELK1, whereas the administration of U0126 effectively inhibited ERK activation and nuclear translocation of ELK1 (Fig. 5b). In addition, we observed a reduced ELK1 expression outside the nucleus following ERK activation (Fig. 5b). In contrast, ERK activation caused increased p-ELK1 expression in the nucleus (Fig. 5b). Immunofluorescence revealed an increased nuclear ELK1 expression upon exposure to CCL2 (Fig. 5c). These results suggest that ELK1 undergoes phosphorylation and is transported to the nucleus in GBC cells, which is facilitated by ERK activation.

To investigate the regulatory role of ELK1 on SNAIL, we identified nine binding sites for both factors using JASPAR [44] (Supplementary Table 8). Subsequently, we constructed plasmids for ELK1 overexpression and plasmids containing full-length and truncated fragments of the SNAIL promoter. Transfection of the ELK1 overexpression plasmid and the plasmid containing the full-length fragment of the SNAIL promoter increased luciferase activity in GBC cells. This increase was greater than that observed when only the ELK1 overexpression plasmid and the truncated fragment of the SNAIL promoter were transfected (Fig. 5d). The binding site of the ELK1 on SNAIL promoter was between positions − 1430 and − 830 (Fig. 5d). The two binding sites were changed to Mut-1 (-1320 to -1329) and Mut-2 (-1143 to -1152). Co-transfection of GBC cells with an ELK1 overexpression and Mut-1 plasmids caused a significant increase in relative luciferase activity compared with those of the control and Mut-2 plasmids. This suggests that ELK1 specifically binds to the Mut-2 site of SNAIL (Fig. 5e). Furthermore, CHIP analysis confirmed that ELK1 regulates SNAIL expression by binding to its sequence, -1143–-1152 (Fig. 5f and g).

### CCL2 triggers augmented stemness in GBC cells through the MEK/ERK/ELK1/SNAIL pathway

**Fig. 6:**
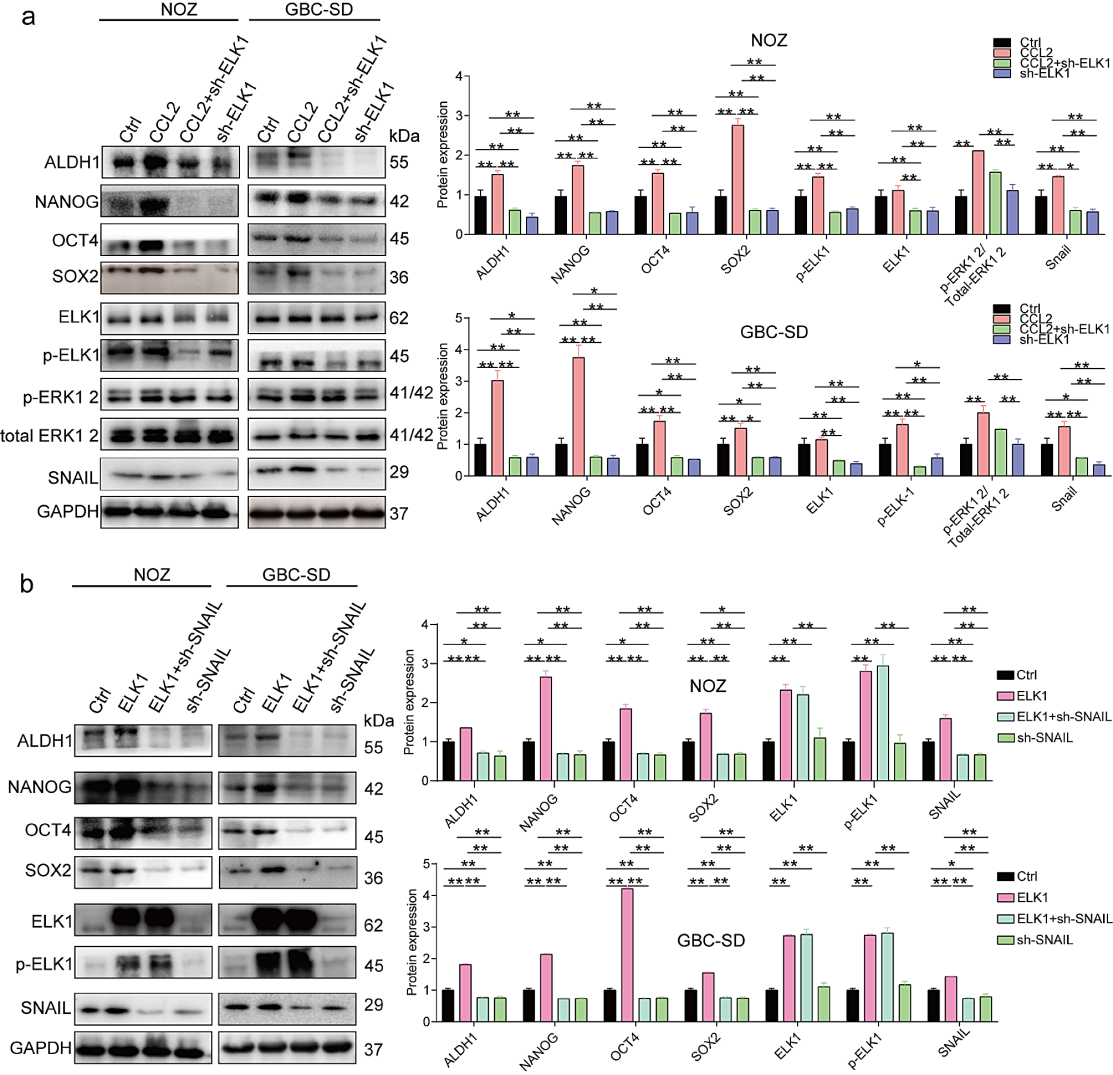
CCL2 triggers augmented stemness in GBC cells through the MEK/ERK/ELK1/SNAIL pathway (**a**) Western blotting was performed to determine the expression of ELK1, p-ELK1, ERK, p-ERK, SNAIL, and CSC markers in GBC cells (*n* = 3). (**b**) Western blotting was conducted to determine the expression of ELK1, p-ELK1, SNAIL, and CSC markers in GBC cells (*n* = 3). Data are presented as mean ± SD. GBC: gallbladder cancer. Statistical significance was assessed using the Student’s *t-test* (a, b). *P < 0.05, **P < 0.01

We further validated the regulation of GBC cell stemness by ELK1/SNAIL. ERK/ELK1 activation in GBC cells caused the upregulation of ALDH1, NANOG, SOX2, and OCT4 (Fig. 6a; Supplementary Fig. 7d and e show the validation of ELK1 overexpression and knockdown). Conversely, ELK1 knockdown hindered the elevation of these proteins (Fig. 6a). Similarly, ELK1 knockdown caused a decreased p-ELK1 expression, impeding the nuclear translocation of ELK1 and the regulation of SNAIL (Fig. 6a), leading to a reduced SNAIL expression (Fig. 6a). In contrast, ELK1 upregulation caused increased expression levels of ALDH1, NANOG, SOX2, and OCT4 (Fig. 6a). In contrast, SNAIL knockdown abrogated the effects of ELK1 overexpression and reduced the stemness of GBC cells (Fig. 6b, Supplementary Fig. 7econfirms SNAIL degradation). These suggest that CCL2-mediated regulation of SNAIL expression through MEK/ERK/ELK1 contributes to the improvement of stemness in GBC cells.

### Promotion of M2-like TAMs by GBCSCs

**Fig. 7:**
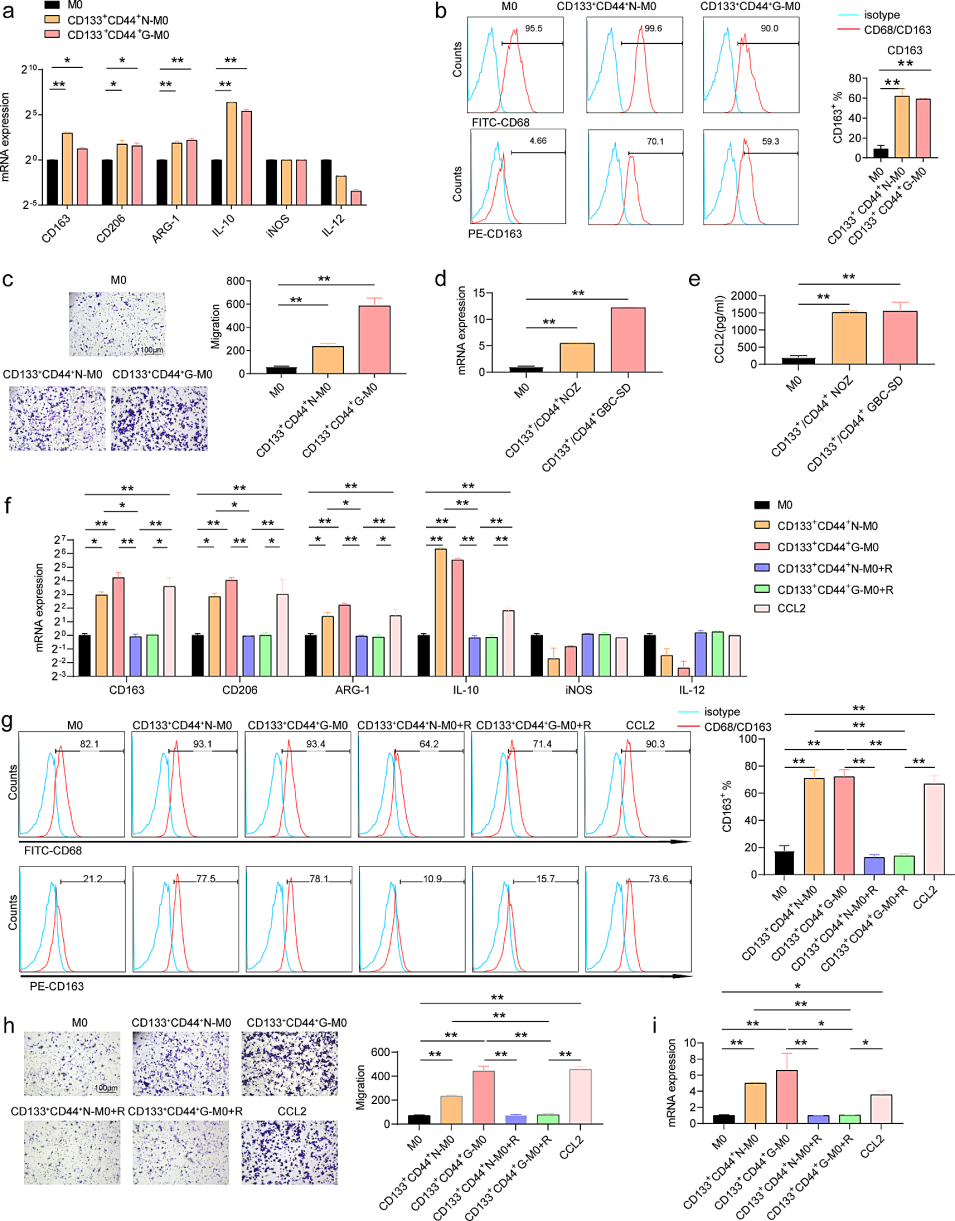
Promotion of M2-like TAMs by GBCSCs. (**a**) qPCR was performed to evaluate CD163, CD206, ARG-1, IL-10, INOS, and IL-12 expression in M0 (*n* = 3). (**b**) Flow cytometry was used to measure the expression of CD68 and CD163 in M0 (*n* = 3). (**c**) Migration of M0 (*n* = 3). (**d**) qPCR was used to determine CCL2 expression in M0 co-cultured with GBCSCs (*n* = 3). (**e**) ELISA was used to determine CCL2 secretion from M0 co-cultured with GBCSCs (*n* = 3). (**f**) qPCR was performed to evaluate the expression of M2-like TAM markers in M0 (*n* = 3). (**g**) Flow cytometry was used to measure CD68 and CD163 expression in M0 (*n* = 3). (**h**) Migration of M0 (*n* = 3). (**i**) qPCR was performed to determine CCL2 expression in M0 (*n* = 3). Data are presented as mean ± SD. ELISA: Enzyme-linked immunosorbent assay, GBCSCs: gallbladder cancer stem cells. Statistical significance was assessed using the Student’s *t-test* (a–i). *P < 0.05, **P < 0.01

The above information suggests that M2-like TAMs may promote GBCSCs (Fig. 1). A positive correlation was observed among the expression of CD163, CD68, CD44, and CD133 in the TME (Supplementary Fig. 2a), and a positive feedback loop may exist between M2-like TAMs and GBCSCs. To further investigate this connection, we performed a co-culture experiment with M0 macrophages and GBCSCs isolated through fluorescence-activated Cell Sorting based on CD133 and CD44 expression. Notably, the expression levels of M2-like TAM markers, including ARG-1, IL-10, CD206, and CD163, were significantly increased (Fig. 7a). In contrast, no significant differences were observed in the levels of TAMs-M1 markers, including iNOS and IL-12(Fig. 7a). Moreover, the co-culturing resulted in an increased proportion of CD68^+^ CD163^+^ M2-like TAMs within the M0 macrophage population (Fig. 7b). This suggests that M0 macrophages undergo alternative activation in response to GBCSCs. Furthermore, co-culturing with GBCSCs caused increased migration of M0 macrophages (Fig. 7c). To further investigate the effects of co-culturing with GBCSCs, we examined the expression levels of CCL2 in M0 macrophages. Co-culturing with CD133^+^ CD44^+^ GBCSCs caused stimulated secretion of CCL2 in M0 macrophages (Fig. 7d and e). RNA-seq of GBC cells co-cultured with M2-like TAMs revealed a significant upregulation of CCL2 expression (Supplementary Fig. 8a). The CCL2-CCR2 signaling pathway is crucial in the recruitment and polarization of M2-like TAMs [21]. To further validate the influence of CCL2 on macrophage recruitment, polarization, and secretion, we used RS504393. Our results suggest that RS504393 effectively inhibited the expression of M2-like TAM markers (Fig. 7f and g). RS504393 reduced the migration of M0 macrophages (Fig. 7h). Additionally, qPCR confirmed that RS504393 inhibited the ability of GBCSCs to promote CCL2 secretion by macrophages (Fig. 7i). The results show that through CCL2 secretion, GBCSCs enhances the migration of macrophages, leading to alternative polarization and further secretion of CCL2. Consequently, a positive feedback loop is established between GBCSCs and M2-like TAMs in the TME of GBC, where CCL2 plays a central role.

## Targeted inhibition of CCL2 to treat GBC

**Fig. 8:**
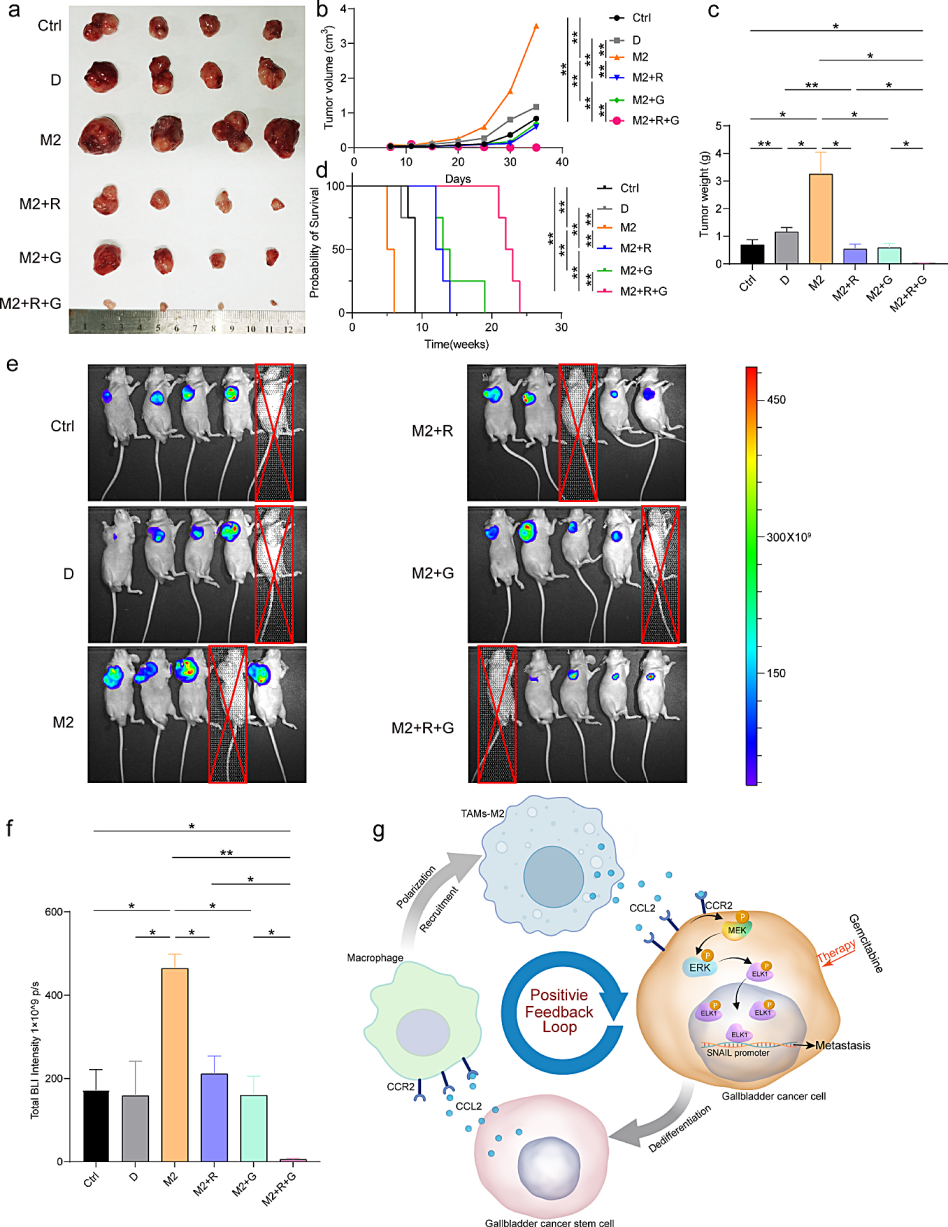
Targeted inhibition of CCL2 to treat GBC. (**a**) Images of subcutaneous tumors (*n* = 4). (**b**) Growth curves of subcutaneous tumors (*n* = 4). (**c**) Weight of subcutaneous tumors (*n* = 4). (**d**) Survival of nude mice (*n* = 4). (**e**) BLI of subcutaneous tumors. Mice were anesthetized to death (*n* = 4). (**f**) Total BLI intensity of live imaging (*n* = 4). (**g**) Positive feedback between GBCSCs and TAMs-M2. Data are presented as mean ± SD. BLI: bioluminescence, D: Dissolution agent, GBC: gallbladder cancer, M2:M2-Like tumor-associated macrophage, R: RS504393, G: gemcitabine. Statistical significance was assessed using the Student’s *t-test* (b, c, and e) and log-rank (d). *P < 0.05, **P < 0.01

CCL2 secretion by M2-like TAMs promotes EMT, stemness, and metastasis of GBC. Additionally, GBCSCs enhanced CCL2 secretion via M2-like TAMs. The positive feedback loop between CCL2 and M2-like TAMs can be disrupted through targeted inhibition of CCR2. To assess the therapeutic effects of combining targeted CCR2 inhibition with gemcitabine treatment in GBC, a subcutaneous tumor model was created and treated with RS504393 and gemcitabine. The presence of M2-like TAMs contributed to the increased size and weight of the subcutaneous tumors (Fig. 8a–c). Administration of RS504393 or gemcitabine as individual therapies showed a therapeutic effect on the tumor (Fig. 8a–c). However, when these therapies were combined, a significant reduction in the subcutaneous tumor size was observed (Fig. 8a–c). Survival experiments revealed that untreated mice did not survive beyond Week 10, whereas mice that received the monotherapy died by Week 15 (Fig. 8d). In contrast, the combination treatment resulted in a significant improvement in survival rates and extended the lifespan of mice to over 20 weeks (Fig. 8d). In vivo BLI was used to confirm that the drug dissolution agent did not affect the total fluorescence intensity (Fig. 8e and f). Furthermore, M2-like TAMs increased the total BLI intensity of subcutaneous tumors (Fig. 8e and f). Nevertheless, co-administration of RS504393 and gemcitabine caused an even more significant attenuation than treatment with either agent alone (Fig. 8e and f). Therefore, gemcitabine combined with CCL2 inhibition can be used to effectively treat GBC, representing a promising treatment approach for patients with GBC.

## Discussion

M2-like TAMs are implicated in many processes associated with tumor progression, including oncogenesis, proliferation, metastasis, immunosuppression, and EMT through the expression of cytokines, chemokines, growth factors, and protein hydrolases [45, 46]. This transition allows tumor cells to adopt a more metastatic and invasive phenotype and stemness [47]. The self-renewal characteristics of CSCs can induce tumorigenesis, causing metastasis and recurrence and contributing to the attraction of TAMs [48]. In this study, we investigated mutual interactions between GBCSCs and M2-like TAMs. GBCSCs are crucial in attracting macrophages to the GBC TME and in inducing polarization. Additionally, we investigated the mechanisms by which M2-like TAMs promote EMT, stemness, and metastasis in GBC.

TAMs exhibit two polarization states. M2-like TAMs are anti-inflammatory macrophages and facilitate tumor progression [49]. The cytokines released by M2-like TAMs contribute to enhanced EMT and stemness in tumor cells, promoting malignant tumor progression [50, 51]. In GBC cells, DLGAP5 promotes macrophage proliferation, migration, and M2 polarization, driving tumor progression [52]. Furthermore, GBC cell-derived exosomes induce macrophage polarization, facilitating GBC cell invasion and metastasis [53]. The exact mechanism by which M2-like TAMs affect GBC cells remains unclear; however, there is a positive association between the M2-like TAM markers CD68 and CD163 and the GBCSC markers CD44 and CD133 in the tissues of patients with GBC. GBCSCs promote the polarization of macrophages toward M2-like TAMs in a co-culture system. The M2-like TAMs, in turn, enhance EMT in GBC cells, causing their dedifferentiation into more aggressive GBCSCs and increased migration. These led to a positive feedback loop between GBCSCs and M2-like TAMs in GBC.

Owing to the EMT, tumor cells exhibit enhanced migratory and invasive properties. These changes are closely related to invasion, migration, metastasis, and the acquisition of stem cell-like characteristics [54]. CSCs are significant in promoting tumor metastasis, resistance to therapeutic drugs, and recurrence [55]. CSCs exhibit a nonadherent growth pattern and spread to different anatomical sites, facilitating the development of secondary tumors [56]. Conversely, CSCs can contribute to metastasis by promoting the formation of new blood vessels and the secretion of pro-angiogenic factors. In previous studies, the presence of EMT in GBC was significantly associated with invasion and metastasis [57]. Furthermore, OCT4, CD133 [58], CD44 [59], SOX2, NANOG, and ALDH1 [60] are associated with poor patient survival and prognosis in GBC. Our results suggest that M2-like TAMs enhance EMT, de-differentiation into GBCSCs, and migration of GBC cells. M2-like TAMs can produce CCL2 to activate the MEK/ERK signaling pathway and promote the phosphorylated nuclear translocation of ELK1 in GBC cells. The binding of ELK1 to the SANIL promoter in the nucleus leads to an increase in its expression. Consequently, our study highlights the significance of M2-like TAMs in the malignant progression of GBC (Fig. 8g).

The TME is a complex milieu comprising various cellular and molecular components. M2-like TAMs represent a crucial subset of immune cells in the TME. The development of novel therapeutic modalities to remodel the local immune microenvironment can enhance the therapeutic efficacy [61, 62]. The CCL2-CCR2 signaling pathway plays a crucial role in tumorigenesis and has various functions in the progression of malignant diseases [63]. CCL2 acts as an extracellular signal and facilitates migration, invasion, and survival in cancers such as breast cancer [64], prostate cancer [65], and hepatocellular carcinoma [66]. Surgery remains the primary approach for treating GBC because of the lack of effective pharmacological treatments. Notably, CCL2 is significant in the interactions between M2-like TAMs and GBC cells. RS504393 was used to eliminate GBCSCs in vivo. The results of this study show that the combination of this drug significantly inhibited the growth of GBC xenograft tumors. Thus, this study presents a valuable therapeutic target for targeted GBC therapy and provides a new therapeutic approach for GBC.

Acknowledging a limitation in our study is essential. The sample size of patients with GBC was relatively small, which might limit the generalizability of our findings. Despite using a rigorous methodological approach, the constrained sample size hindered an efficient identification of smaller effect sizes, which could impact the statistical power of our analysis. Future research with a larger sample size is essential to validate our results and delve deeper into the intricacies of our observations across a wider patient population. Notably, the insights from this study are critical for advancing targeted therapies and personalized treatment approaches for GBC, underscoring the significance of M2-like TAMs in new therapeutic strategies aimed at disrupting GBC stemness and curbing metastasis. However, it should also be recognized that GBC is a malignancy characterized by high cellular heterogeneity. The pronounced cellular heterogeneity within GBC underscores the challenge of achieving widespread and effective treatment outcomes using single-target drugs or combined therapies, which should be considered when interpreting and applying our findings.

## Conclusions

This study provides evidence for the existence of a positive feedback loop between M2-like TAMs and GBCSCs, with CCL2 playing a pivotal role. CCL2 modulates SNAIL expression by facilitating the translocation of ELK1 into the nucleus via MEK/ERK phosphorylation. Subsequently, SNAIL promotes the expression of stemness and EMT markers in GBC cells and improves metastasis and solid tumor growth in GBC. Encouraging therapeutic results have been observed following the combined administration of gemcitabine and the CCL2 receptor antagonist RS504393 in patients with GBC. Our study identified a promising novel target for therapeutic intervention in GBC.

### Electronic supplementary material

Below is the link to the electronic supplementary material.

Supplementary Material 1

Supplementary Material 2

## Data Availability

Publicly available datasets were analyzed in this study. The data can be found https://dataview.ncbi.nlm.nih.gov/object/PRJNA1048112?reviewer=jpecu1mphh4l9nh0dnb429nnmq and Proteomics uploading.The mass spectrometry proteomics data have been deposited to the ProteomeXchange Consortium (https://proteomecentral.proteomexchange.org) via the iProX partner repository with the dataset identifier PXD050842.

## Abbreviations

CSCs: Cancer stem cells
CCL2: Chemotactic Cytokines Ligand 2
CCR2: Chemokine (C-C motif) receptor 2
EMT: Epithelial-mesenchymal transformation
EMT-TFs: EMT-inducing transcription factors
ERK: Extracellular-regulated protein kinases
GBC: Gallbladder cancer
GBCSCs: Gallbladder cancer stem cells
MEK: Mitogen-activated protein kinase kinase
PMA: Phorbol 12-myristate 13-acetate
TAMs: Tumor-associated macrophage
TME: Tumor microenvironment
