# Supplementary material for: M2-like tumor-associated macrophage-secreted CCL2 facilitates gallbladder cancer stemness and metastasis
Source: Exp Hematol Oncol. 2024 Aug 13;13:83. doi: 10.1186/s40164-024-00550-2 (PMC11320879; doi:10.1186/s40164-024-00550-2)
Supplement: Supplementary file 1 — Supplementary Material 1 [file 40164_2024_550_MOESM1_ESM.docx]

**Supplementary Figures and Legends**


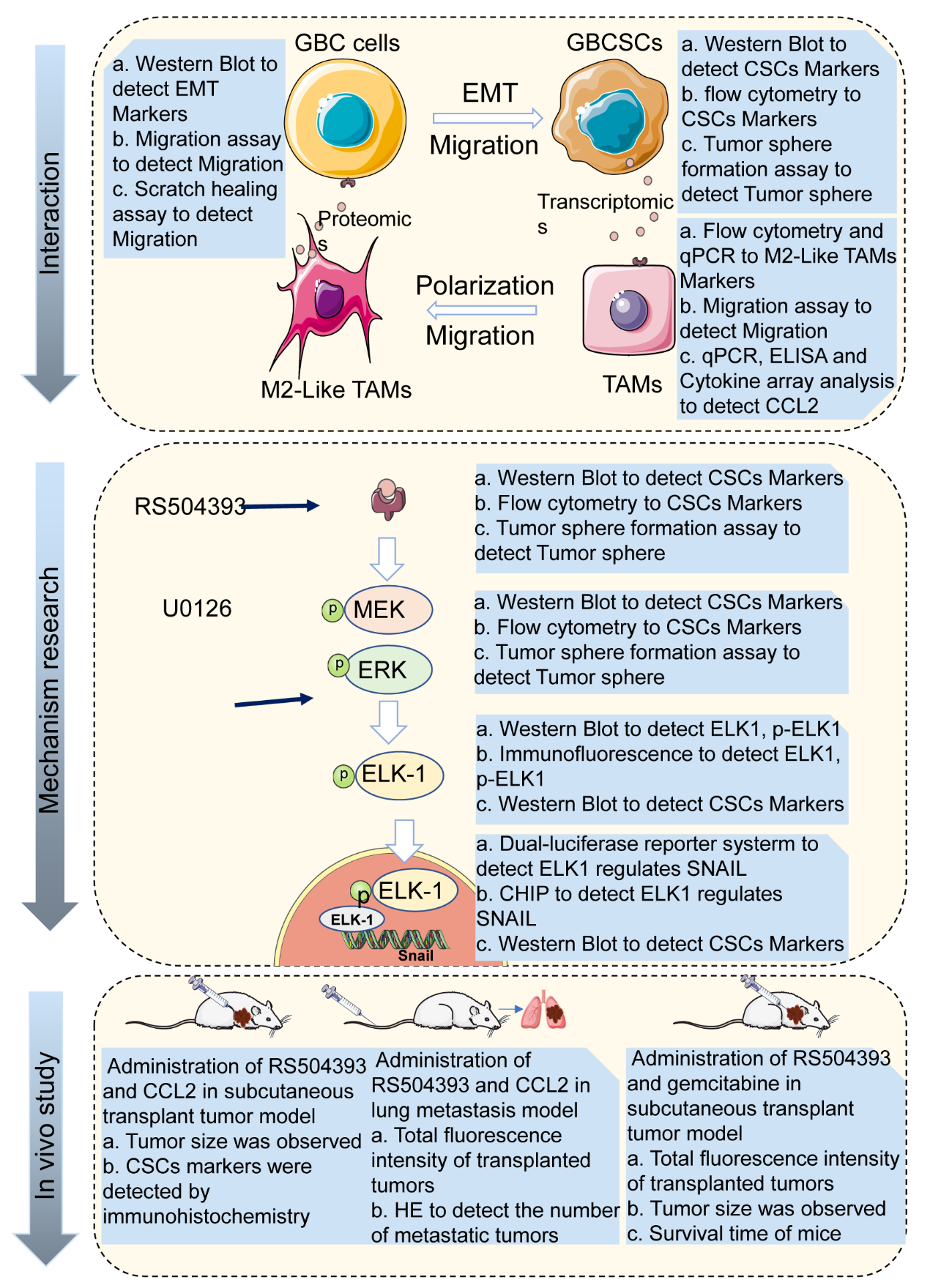


Supplementary Fig.1 Flow diagram of the study.


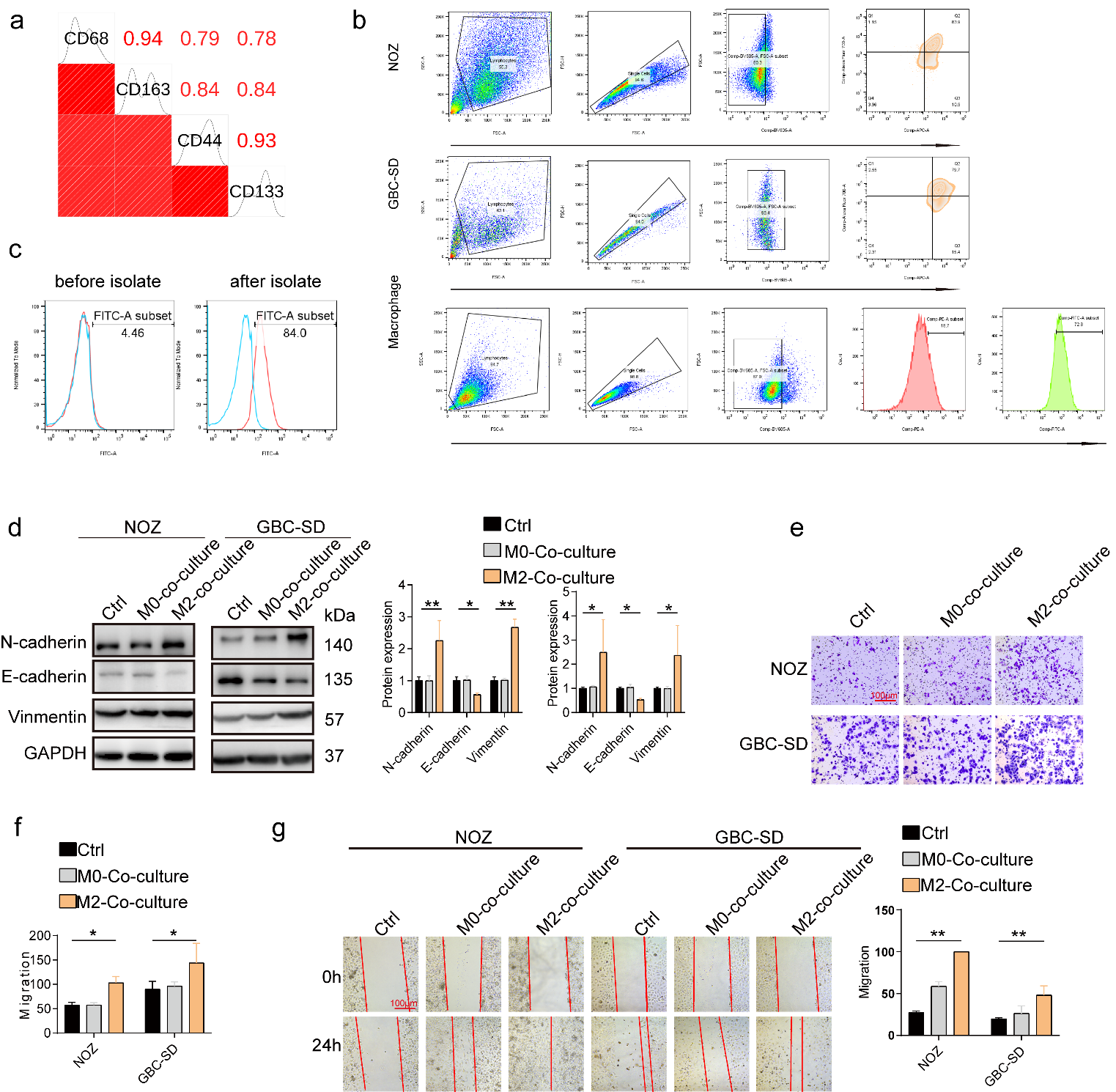


Supplementary Fig.2 Validation of Knockdown Overexpression and migration of GBC cells

(**a**) The correlation of CD163, CD68, CD44 and CD133 expression in 24 samples of GBC tissues. (**b**) Logic gate for drawing NOZ GBC-SD macrophages. (**c**) Validation of CD14^+^ before and after magnetic bead sorting. (**d**) Western blotting for N-Cadherin, E-Cadherin and Vimentin expression in GBC cells(*n*=3). (**e**) Trans-well chamber migration assay to detect migration in gallbladder cancer cells(*n*=3). (**f**) Statistical analysis of migrated cells(*n*=3). (**g**)Wound healing assay of GBC cells (*n*=3). Data are presented as mean ± SD. GBC: gallbladder cancer, M2:M2-like tumor-associated macrophage. Statistical significance was assessed by Pearson correlation (a) and Student’s *t-test* (d, e, g). **P*<0.05, ***P*<0.01.


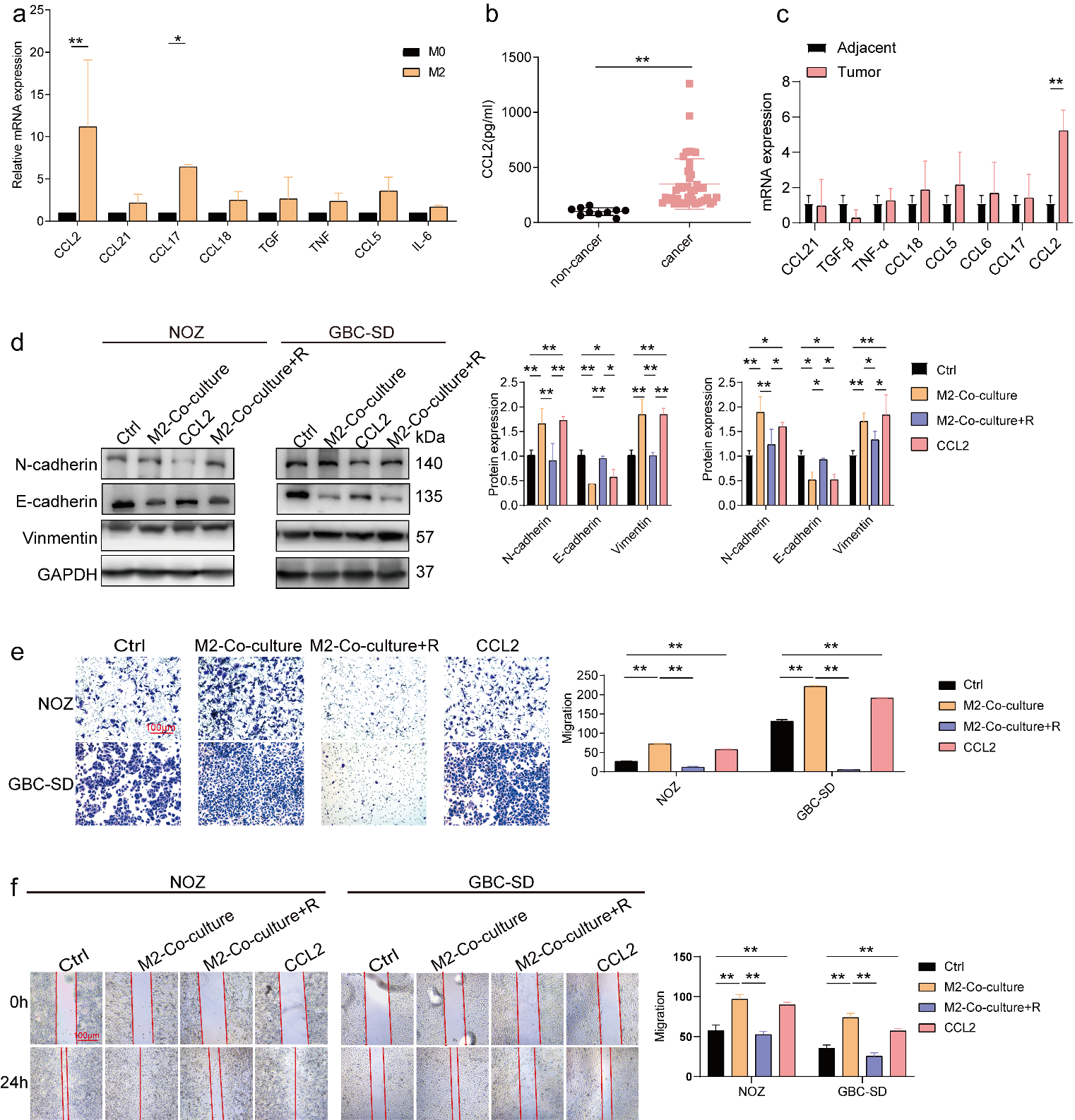


Supplementary Fig.3 Increased CCL2 expression in M2-like TAMs and migration of GBC cells

(**a**) qPCR was performed to identify differences in cytokine expression in M0 and M2-like TAMs (*n*=3). (**b**) ELISA was used to detect CCL2 levels in the serum of patients with GBC, gallbladder stones or polyps (*n*=3). (**c**) qPCR was performed to determine differences in cytokine expression in macrophages extracted from tissue samples from patients with GBC (*n*=3). (**d**) Western blotting was used to measure the expression of N-cadherin, E-cadherin and Vimentin in GBC cells(*n*=3). (**e**) Trans-well chamber migration assay was performed to detect the migration of GBC cells(*n*=3). (**f**) Wound healing assay was used to determine the migration ability of GBC cells(*n*=3). Data are presented as mean ± SD. GBC: gallbladder cancer, M2: M2-Like tumor-associated macrophage, R: RS504393. Statistical significance was assessed by Student’s *t-test* (a-f). **P*<0.05, ***P*<0.01.


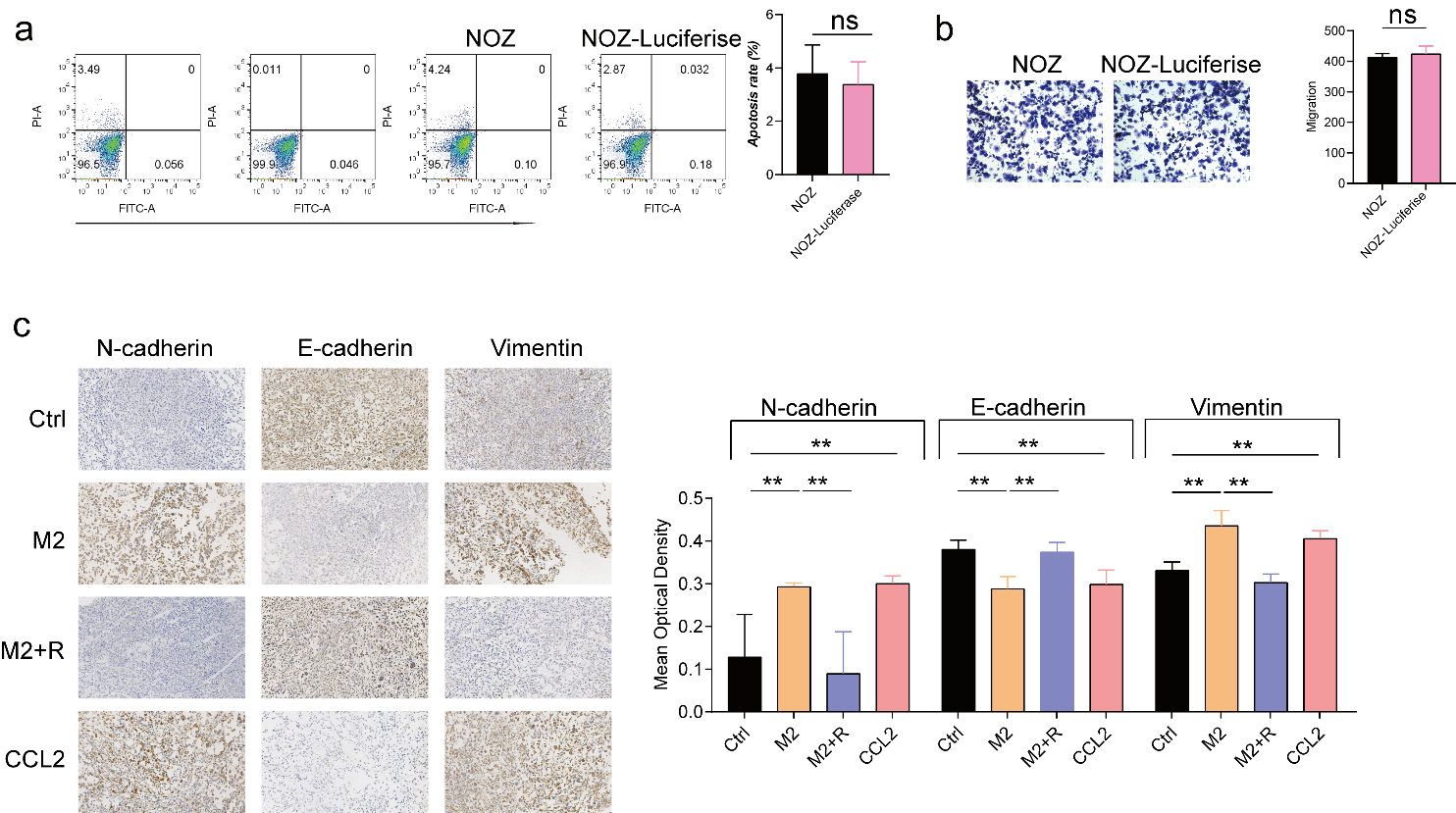


Supplementary Fig.4 Promotion of EMT and Apoptosis of NOZ-Luciferase by M2-like TAMs via CCL2 secretion in GBC in vivo

(**a**) Flow cytometry to detect NOZ and NOZ-Luciferase apoptosis (*n*=3). (**b**) Migration assay to check the migration of NOZ and NOZ-Luciferase (*n*=3). (**c**) Immunohistochemistry was performed to determine the expression of N-cadherin, E-cadherin, and Vimentin in subcutaneous tumors(*n*=3). Data are presented as mean ± SD. GBC: gallbladder cancer, M2: M2-Like tumor-associated macrophage, R: RS504393. Statistical significance was assessed by Student’s *t-test* (a-c). **P*<0.05, ***P*<0.01.


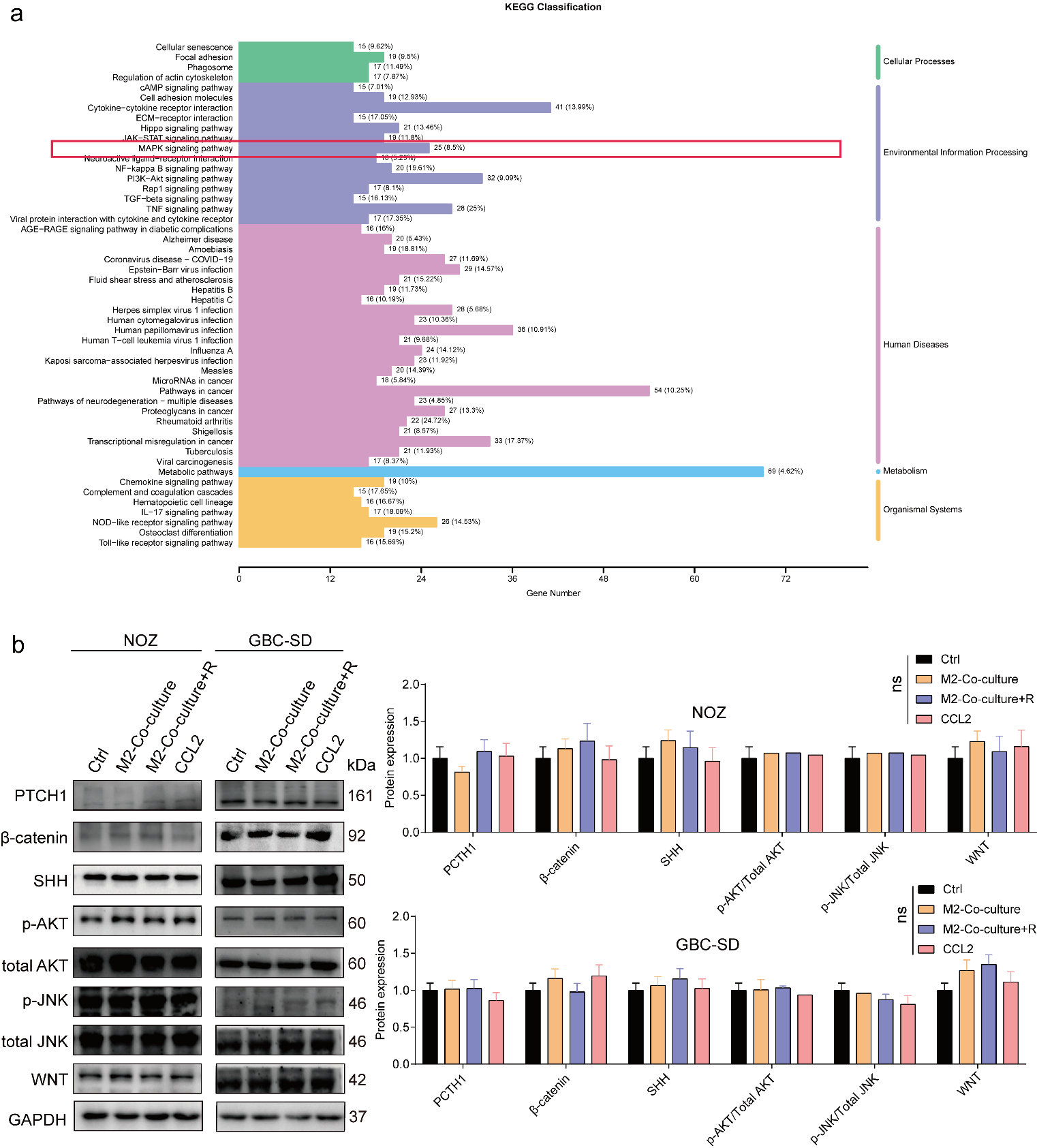


Supplementary Fig.5 M2-like TAMs secreted CCL2 to activate MEK in GBC cells

(**a**) Pathway enrichment analysis of differentially expressed genes from transcriptome sequencing (*n*=3). (**b)** Western blotting for PTCH1, WNT, β-catenin, p-AKT, total-AKT, SHH, p-JNK and total-JNK expression in GBC cells(*n*=3). Data are presented as mean ± SD. GBC: gallbladder cancer, M2:M2-like tumor-associated macrophage, R: RS504393. Statistical significance was assessed by Student’s *t-test* (a-b). **P*<0.05, ***P*<0.01.


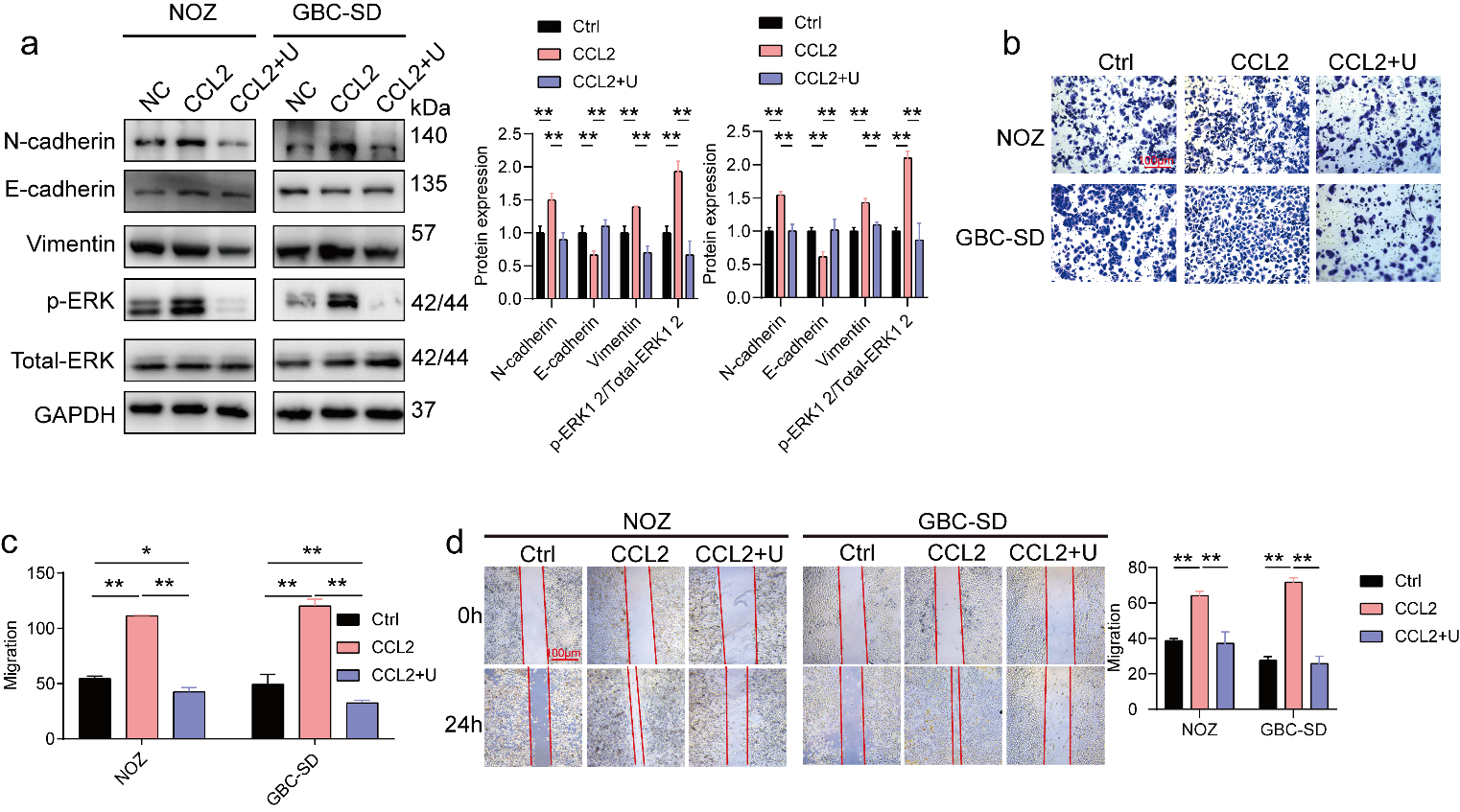


Supplementary Fig.6 The promotion of EMT GBC and MEK/ERK migration by CCL2. Fig

(**a**) Western blotting was used to measure the expression of N-cadherin, E-cadherin and Vimentin in GBC cells(*n*=3). (**b**) Representative images of the Trans-well chamber migration assay in GBC cells. (**c**) Statistical analysis of migrated cells(*n*=3). (**d**) Wound healing assay to detect migration of GBC cells migration(*n*=3). Data are presented as mean ± SD. GBC: gallbladder cancer, U: U0126. Statistical significance was assessed by Student’s *t-test* (a, c, d). **P*<0.05, ***P*<0.01.


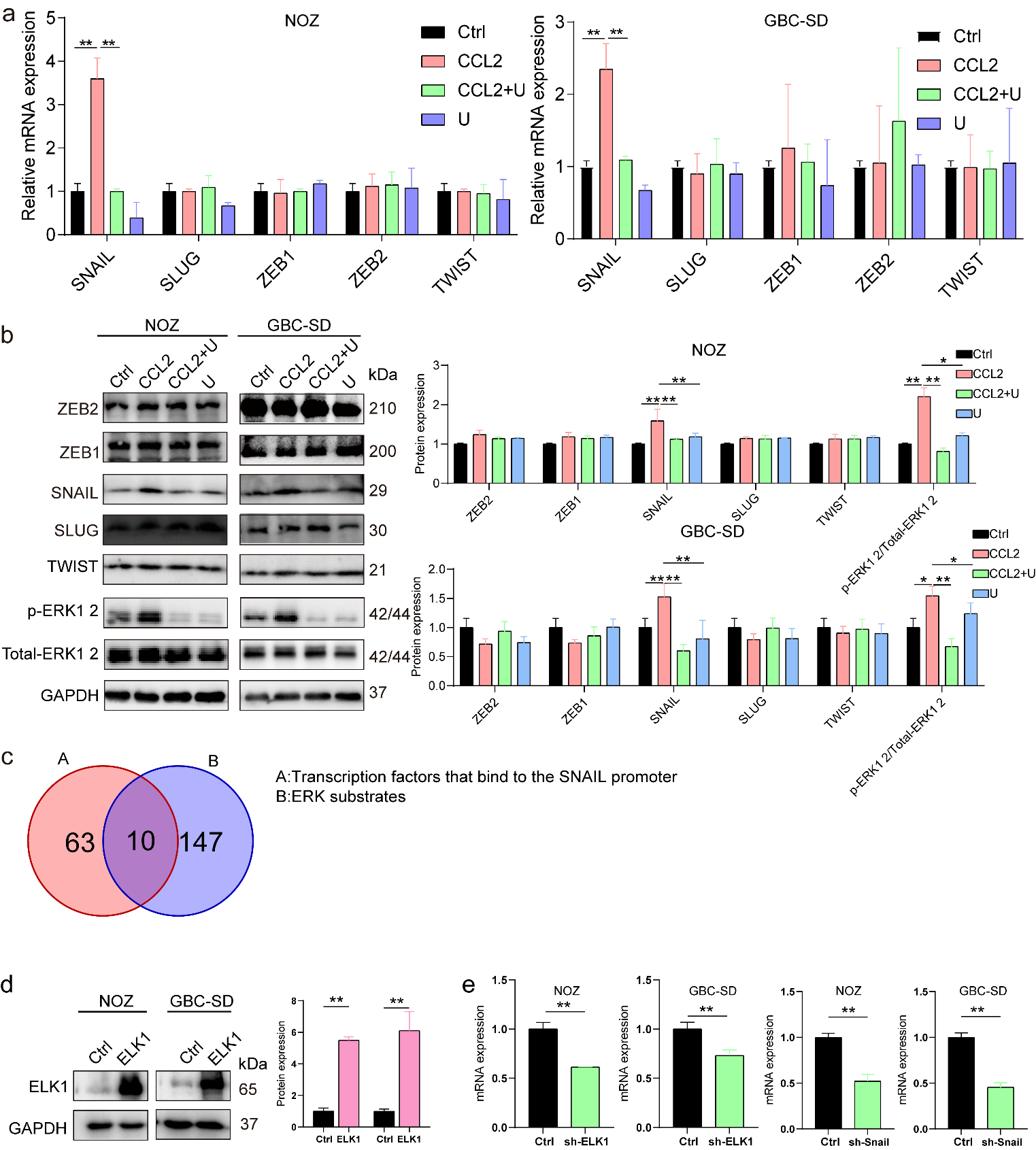


Supplementary Fig.7 CCL2 activates the MEK/ERK/ELK1/SNAIL signaling pathway in GBC cells

(**a**) qPCR detection of SNAIL, SLUG, ZEB1, ZEB2 and TWIST expression in GBC cells (*n* = 3), (**b**) Western blotting detection of SNAIL, SLUG, ZEB1, ZEB2 and TWIST expression in GBC cells (*n* = 3). (**c**) Downstream of ERK with the ability to bind SNAIL promoter transcription factors crossing. (**d**) ELK1, SNAIL knockdown validation in GBC cells (*n*=3). (**e**) ELK1 overexpression validation in GBC cells (*n*=3). Data are presented as mean ± SD. GBC: gallbladder cancer, U: U0126. Statistical significance was assessed by Student’s *t-test* (a, b, d, e). **P*<0.05, ***P*<0.01.


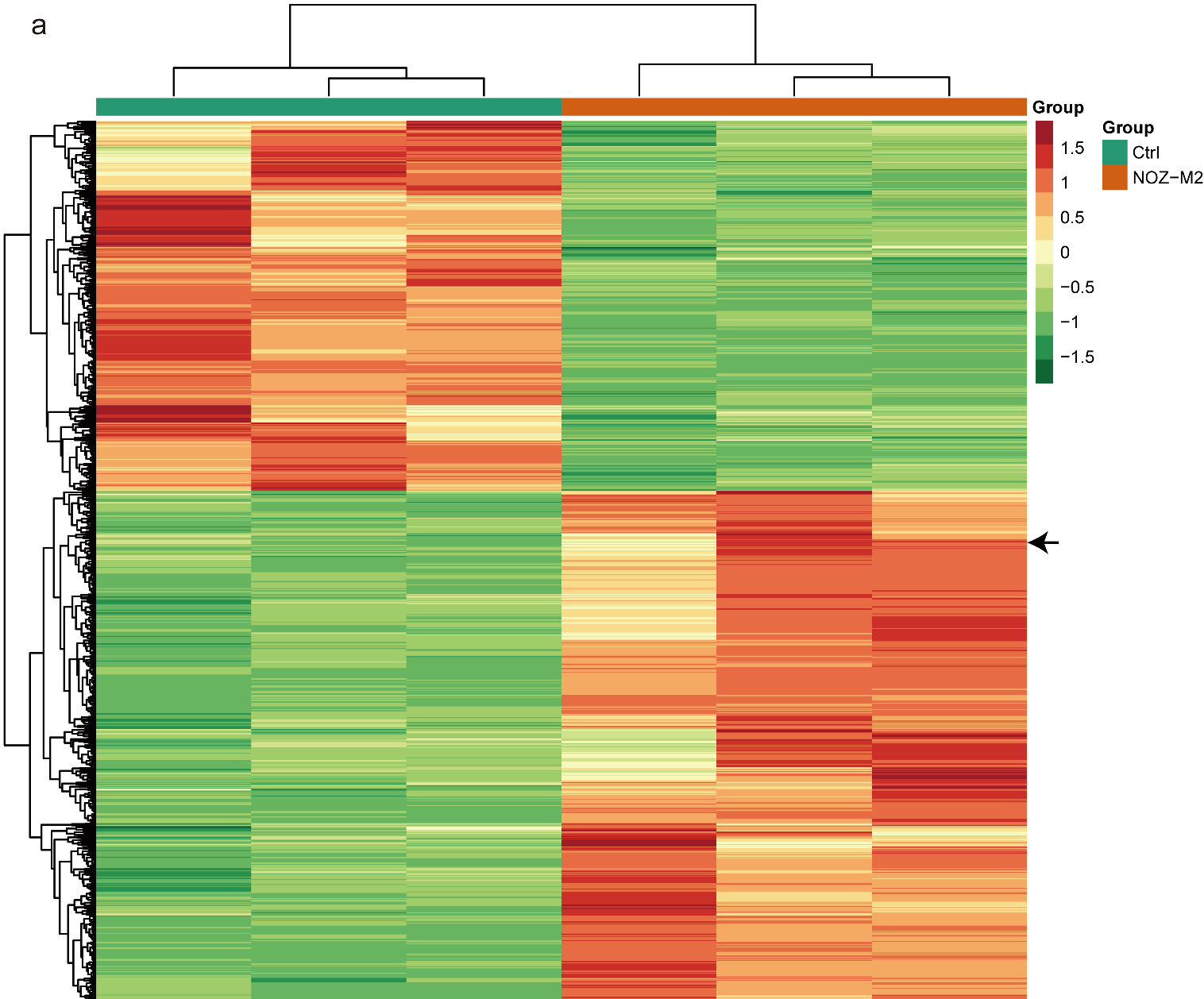


Supplementary Fig.8 Transcriptome sequencing of differential genes in GBC cells co-cultured with M2-like TAMs (*n*=3). Data are presented as mean ± SD. GBC: gallbladder cancer, M2:M2-Like tumor-associated macrophage. Statistical significance was assessed by Student’s *t-test*.

**Supplementary Tables
Supplementary Table 1 The clinical information of the 24 gallbladder cancers**

| Case NO. | Gender | Age | TNM stage | Survival time (month) | Survival state |
| --- | --- | --- | --- | --- | --- |
| 1 | Male | 74 | II | 29.27 | Dead |
| 2 | Female | 75 | IVB | 10.43 | Dead |
| 3 | Male | 56 | IIIA | 42.43 | Dead |
| 4 | Female | 47 | II | 60 | Alive |
| 5 | Female | 53 | IVB | 15.47 | Dead |
| 6 | Female | 66 | IVA | 21.9 | Dead |
| 7 | Female | 70 | IIIA | 15.33 | Dead |
| 8 | Male | 63 | IIIB | 19.4 | Dead |
| 9 | Male | 67 | IVB | 12.37 | Dead |
| 10 | Male | 61 | II | 19.57 | Dead |
| 11 | Female | 48 | IA | 34.70 | Dead |
| 12 | Male | 74 | IVB | 11.43 | Dead |
| 13 | Female | 59 | IIIA | 38.17 | Dead |
| 14 | Female | 43 | IIIA | 39.00 | Dead |
| 15 | Female | 65 | IB | 58.03 | Dead |
| 16 | Male | 68 | IVA | 21.23 | Dead |
| 17 | Female | 61 | II | 35.27 | Dead |
| 18 | Female | 70 | II | 20.17 | Dead |
| 19 | Male | 59 | IIIB | 25.17 | Dead |
| 20 | Male | 47 | II | 49.93 | Dead |
| 21 | Male | 69 | IB | 29.43 | Dead |
| 22 | Male | 77 | IA | 17.3 | Dead |
| 23 | Male | 31 | IA | 60 | Alive |
| 24 | Female | 48 | IA | 60 | Alive |

**Supplementary Table 2 The clinical information of the 8 gallbladder cancer**

| Case NO. | TNM stage | Gender | Age (years) |
| --- | --- | --- | --- |
| 1 | II | Female | 62 |
| 2 | IIB | Female | 76 |
| 3 | IIIA | Male | 66 |
| 4 | IV | Female | 74 |
| 5 | IA | Female | 36 |
| 6 | II | Male | 40 |
| 7 | II | Male | 71 |
| 8 | IB | Male | 68 |

**Supplementary Table 3 The list of antibodies**

| Antibodies | Application | | Dilution | | Cate number | | Source |  |  |
| --- | --- | --- | --- | --- | --- | --- | --- | --- | --- |
| Anti-ELK1 antibody | CHIP | | 1:20 | | ab125085 | | Abcam |  |  |
| Normal Rabbit IgG | CHIP | | 1:20 | | 2729 | | CST |  |  |
| CD44 APC G44-26 | FACS | | 1:20 | | 559942 | | BD |  |  |
| CD133 BV421 293C3 | FACS | | 1:5 | | 566595 | | BD |  |  |
| BV605 Annexin V | | Flow Cyt | 1:20 | | 563974 | | BD | | |
| FITC anti-mouse CD68 Antibody | | Flow Cyt | 1:20 | | 137006 | | BioLegend | | |
| PE anti-human CD163 Antibody | | Flow Cyt | 1:20 | | 333606 | | BioLegend | | |
| Alexa Fluor® 700 anti-human CD44 Antibody | | Flow Cyt | 1:20 | | 397522 | | BioLegend | | |
| APC anti-human CD133 Antibody | | Flow Cyt | 1:20 | | 397906 | | BioLegend | | |
| p-ELK1 Antibody | IF | | 1:100 | | sc-8406 | | Santa Cruz |  |  |
| Recombinant Anti-ELK1 antibody | IF | | 1:100 | | ab32106 | | Abcam |  |  |
| Donkey F(ab')2 Anti-Rabbit IgG H&L (PE) | IF | | 1:200 | | ab7007 | | Abcam |  |  |
| Donkey Anti-Mouse IgG H&L (Alexa Fluor® 488) | IF | | 1:200 | | ab150105 | | Abcam |  |  |
| CD163 Rabbit mAb | IHC | | 1:100 | | 93498 | | CST |  |  |
| CD68 Rabbit mAb | IHC | | 1:100 | | 97778 | | CST |  |  |
| CD44 Mouse mAb | IHC | | 1:100 | | 3570 | | CST |  |  |
| CD133 XP® Rabbit mAb | IHC | | 1:100 | | 64326 | | CST |  |  |
| Goat Anti-Rabbit IgG H&L (HRP) | IHC | | 1:1000 | | ab6721 | | Abcam |  |  |
| Rabbit Anti-Mouse IgG H&L (HRP) | IHC | | 1:1000 | | ab6728 | | Abcam |  |  |
| Phospho-Elk-1 (Ser383) Antibody | WB | | 1:1000 | | 9181 | | CST |  |  |
| ELK1 Antibody | WB | | 1:1000 | | 9182 | | CST |  |  |
| Anti-ELK1 antibody | WB | | 1:1000 | | ab125085 | | Abcam |  |  |
| Oct-4A (C30A3) Rabbit mAb | WB | | 1:1000 | | 2840 | | CST |  |  |
| Sox2 (D6D9) XP ® Rabbit mAb | WB | | 1:1000 | | 3579 | | CST |  |  |
| ALDH1A1 (D9J7R) XP ® Rabbit mAb | WB | | 1:1000 | | 36671 | | CST |  |  |
| Nanog (D73G4) XP ® Rabbit mAb | WB | | 1:1000 | | 4903 | | CST |  |  |
| Wnt5a Antibody | WB | | 1:1000 | | 2392 | | CST |  |  |
| SHH Antibody | WB | | 1:1000 | | 2207 | | CST |  |  |
| PTCH1 Antibody | WB | | 1:1000 | | 2468 | | CST |  |  |
| β-Catenin Antibody | WB | | 1:1000 | | 9562 | | CST |  |  |
| Anti-JNK1 + JNK2 + JNK3 antibody | WB | | 1:1000 | | ab179461 | | Abcam |  |  |
| Anti-JNK1 + JNK2 + JNK3 (phospho T183 + Y185) antibody | WB | | 1:1000 | | ab59196 | | Abcam |  |  |
| Pan-AKT Rabbit mAb | WB | | 1:1000 | | 4691 | | CST |  |  |
| Phospho-AKT1-T308+AKT2-T309+AKT3-T305 Rabbit pAb | WB | | 1:1000 | | AP1172 | | ABclone |  |  |
| MEK1/MEK2 Antibody | WB | | 1:1000 | | 9122 | | CST |  |  |
| Phospho-MEK1/2 (Ser217/221) (41G9) Rabbit mAb | WB | | 1:1000 | | 9154 | | CST |  |  |
| Anti -Vinculin Rabbit pAb | WB | | 1:1000 | | 1309 | | CST |  |  |
| Phospho-p44/42MAPK(Erk1/2) (Thr202/Tyr204) (D13.14.4E) XP® Rabbit mAb | WB | | 1:1000 | | 4370 | | CST |  |  |
| p44/42 MAPK (Erk1/2) (137F5) Rabbit mAb | WB | | 1:1000 | | 4695 | | CST |  |  |
| GAPDH (D16H11) XP® Rabbit mAb | WB | | 1:1000 | | 5174 | | CST |  |  |
| ZEB1 (D80D3) Rabbit mAb | WB | | 1:1000 | | 3396 | | CST |  |  |
| ZEB2 (E6U7Z) Rabbit mAb | WB | | 1:1000 | | 97885 | | CST |  |  |
| Slug (C19G7) Rabbit mAb | WB | | 1:1000 | | 9585 | | CST |  |  |
| TWIST1 (E5G9Y) Rabbit mAb | WB | | 1:1000 | | 90445 | | CST |  |  |
| Snail (C15D3) Rabbit mAb | WB | | 1:1000 | | 3879 | | CST |  |  |
| PARP (46D11) Rabbit mAb | WB | | 1:1000 | | 9532 | | CST |  |  |
| Histone H3 Antibody | WB | | 1:1000 | | 9715 | | CST |  |  |
| E-Cadherin (24E10) Rabbit mAb | WB | | 1:1000 | | 3195 | | CST |  |  |
| N-Cadherin (D4R1H) XP® Rabbit mAb | WB | | 1:1000 | | 13116 | | CST |  |  |
| Vimentin (D21H3) XP® Rabbit mAb | WB | | 1:1000 | | 5741 | | CST |  |  |
| Goat Anti-Rabbit IgG H&L (HRP) | WB | | 1:1000 | | ab205718 | | Abcam |  |  |
| Goat Anti-Mouse IgG H&L (HRP) | WB | | 1:1000 | | ab205719 | | Abcam |  |  |

Notes: CHIP: Chromatin immunoprecipitation, FACS: Fluorescence activated Cell Sorting, Flow cyt: Flow Cytometry, IHC: Immunohistochemistry, IF: Immunofluorescence, WB: Western blotting.

**Supplementary Table 4 The list of primer sequences used for the qPCR**

| Gene | Forward primer (5' to 3') | Reverse primer (5' to 3') |
| --- | --- | --- |
| CCL21 | TCAGGACTGTTGCCTCAAGT | CAGGATAGCTGGGATGGAGC |
| TGF-β | GAGCCCTGGACACCAACTAT | AAGTTGGCATGGTAGCCCTT |
| TNF-α | TCCTCTCTGCCATCAAGAGC | AGTAGACCTGCCCAGACTCG |
| CCL18 | GCTGCCTCGTCTATACCTCC | CCGGCCTCTCTTGGTTAGGA |
| CCL5 | CCTCATTGCTACTGCCCTCT | ATACTCCTTGATGTGGGCACG |
| CCL6 | AAGCCAGAGCTGTGCAGATG | CTGGCATTTGTGGTTGGGTC |
| CCL17 | CAGCTCGAGGGACCAATGTG | CCTGCCCTGCACAGTTACAA |
| CCL2 | GATGCAATCAATGCCCCAGTC | TTTGGGACACTTGCTGCTGG |
| SNAIL | CGGAAGCCTAACTACAGCGA | GCCAGGACAGAGTCCCAGAT |
| SLUG | CAAGGACACATTAGAACTCACAC | CTACACAGCAGCCAGATTCC |
| ZEB1 | CAGCTTGATACCTGTGAATGG | TATCTGTGGTCGTGTGGGACT |
| ZEB2 | GATATGACAGACTCCGACTCCT | CGATAAGGTGGTGCTTGTGTT |
| TWIST | TACGCCTTCTCGGTCTGGAG | TTCTCTGGAAACAATGACATCTAGG |
| CD163 | TTTGTCAACTTGAGTCCCTTCAC | TCCCGCTACACTTGTTTTCAC |
| CD206 | TGGTGAACGGAATGATTGTGTAG | GGTCCATCTTCCTTGTGTCA |
| ARG-1 | TGGACAGACTAGGAATTGGCA | CCAGTCCGTCAACATCAAAACT |
| IL-10 | TCAAGGCGCATGTGAACTCC | GATGTCAAACTCACTCATGGCT |
| CD86 | TACACGGTTACCCAGAACCT | CCGCGTCTTGTCAGTTTCCA |
| iNOS | TCACCTACTTCCTGGACATCAC | GAACTTCCACTTGCTGTACTCTG |
| IL-12 | AGATGTACCAGGTGGAGTTCA | CGGTTCTTCAAGGGAGGATTT |
| GADPH | CACCCACTCCTCCACCTTTGA | TCTCTCTTCCTCTTGTGCTCTTGC |
| β-actin | TGACGTGGACATCCGCAAAG | CTGGAAGGTGGACAGCGAGG |
| CHIP-SNAIL | ACTCTGAAGCAGTTGCCACTTC | AGGGAGACAGACGAAGTAAACAG |

**Supplementary Table 5 Sequences used for knockdown**

| Target | Sequence (5'-3') | Function |
| --- | --- | --- |
| ELK1 | CCCCAGAAGCCATGACTACTA  GGGGTCTTCGGTACTGATGAT | Human ELK1 knockdown |
| SNAIL | GACCCACTCAGATGTCAAGAA  CTGGGTGAGTCTACAGTTCTT | Human SNAIL knockdown |

**Supplementary Table 6 Correlations between CD68 CD163 and clinical-pathological parameters of gallbladder cancer patients**

| Clinicopathologic Parameters | *n* | CD68 expression | | *P* value | CD163 expression | | *p* value |
| --- | --- | --- | --- | --- | --- | --- | --- |
|  |  | Low | High |  | Low | High |  |
| All patients | 24 | 12 | 12 |  | 12 | 12 |  |
| Gender |  |  |  | 0.6843 |  |  | 0.6843 |
| Female | 12 | 7 | 5 |  | 5 | 7 |  |
| Male | 12 | 5 | 7 |  | 7 | 5 |  |
| Age |  |  |  | 0.2138 |  |  | 0.2138 |
| ≤60 | 10 | 7 | 3 |  | 7 | 3 |  |
| >60 | 14 | 5 | 9 |  | 5 | 9 |  |
| Tumor type |  |  |  | 0.5901 |  |  | 0.0932 |
| Others | 4 | 1 | 3 |  | 0 | 4 |  |
| Adenocarcinoma | 20 | 11 | 9 |  | 12 | 8 |  |
| TNM stage |  |  |  | 0.0033 |  |  | 0.2203 |
| Ⅰ-Ⅱ | 12 | 10 | 2 |  | 8 | 4 |  |
| Ⅲ-Ⅳ | 12 | 2 | 10 |  | 4 | 8 |  |
| Tumor size |  |  |  | >0.9999 |  |  | >0.9999 |
| ≤2 cm | 7 | 4 | 3 |  | 4 | 3 |  |
| >2 cm | 17 | 8 | 9 |  | 8 | 9 |  |
| LVI |  |  |  | 0.0028 |  |  | 0.0361 |
| Absence | 14 | 3 | 11 |  | 4 | 10 |  |
| Presence | 10 | 9 | 1 |  | 8 | 2 |  |
| PNI |  |  |  | 0.0995 |  |  | 0.0123 |
| Absence | 13 | 4 | 9 |  | 3 | 10 |  |
| Presence | 11 | 8 | 3 |  | 9 | 2 |  |
| TI |  |  |  | 0.0094 |  |  | 0.4003 |
| Absence | 15 | 4 | 11 |  | 9 | 6 |  |
| Presence | 9 | 8 | 1 |  | 3 | 6 |  |

**Supplementary Table 7 Correlations between CD44 CD133 and clinical-pathological parameters of gallbladder cancer patients**

| Clinicopathologic Parameters | *n* | CD44 expression | | *p*值 | CD133 expression | | *p*值 |
| --- | --- | --- | --- | --- | --- | --- | --- |
|  |  | Low | High | Low | High |  |  |
| All patients | 24 | 11 | 13 |  | 12 | 12 |  |
| Gender |  |  |  | >0.9999 |  |  | 0.6843 |
| Female | 12 | 5 | 7 |  | 5 | 7 |  |
| Male | 12 | 6 | 6 |  | 7 | 5 |  |
| Age |  |  |  | 0.4081 |  |  | 0.6802 |
| ≤60 | 10 | 6 | 4 |  | 4 | 6 |  |
| >60 | 14 | 5 | 9 |  | 8 | 6 |  |
| Tumor type |  |  |  | 0.5963 |  |  | 0.5901 |
| Others | 4 | 1 | 3 |  | 3 | 1 |  |
| Adenocarcinoma | 20 | 10 | 10 |  | 9 | 11 |  |
| TNM stage |  |  |  | 0.4136 |  |  | 0.0391 |
| Ⅰ-Ⅱ | 12 | 7 | 5 |  | 9 | 3 |  |
| Ⅲ-Ⅳ | 12 | 4 | 8 |  | 3 | 9 |  |
| Tumor size |  |  |  | 0.0233 |  |  | 0.0686 |
| ≤2 cm | 7 | 6 | 1 |  | 6 | 1 |  |
| >2 cm | 17 | 5 | 12 |  | 6 | 11 |  |
| LVI |  |  |  | 0.0111 |  |  | 0.0361 |
| Absence | 14 | 3 | 11 |  | 4 | 10 |  |
| Presence | 10 | 8 | 2 |  | 8 | 2 |  |
| PNI |  |  |  | 0.0377 |  |  | 0.4136 |
| Absence | 13 | 3 | 10 |  | 5 | 8 |  |
| Presence | 11 | 8 | 3 |  | 7 | 4 |  |
| TI |  |  |  | 0.2060 |  |  | 0.4003 |
| Absence | 15 | 5 | 10 |  | 6 | 9 |  |
| Presence | 9 | 6 | 3 |  | 6 | 3 |  |

**Supplementary Table 8 Potential ELK1 binding site on SNAIL promoter**

| Name | Score | Relative score | Start | End | Strand | Predicted sequence |  |
| --- | --- | --- | --- | --- | --- | --- | --- |
| ELK1 | 8.84067 | 0.906033642607 | 616 | 625 | - | CAACCTGAAG | |
| ELK1 | 8.80651 | 0.904898662443 | 1596 | 1605 | + | GCGTCGGAAG | |
| ELK1 | 8.03336 | 0.879205605618 | 1320 | 1329 | - | GCCCCGGACA | |
| ELK1 | 8.03336 | 0.879205605618 | 1738 | 1747 | + | GCCCCGGACA | |
| ELK1 | 7.81307 | 0.871885153108 | 1562 | 1571 | - | GGGGCGGAAA | |
| ELK1 | 5.67759 | 0.836586707799 | 1143 | 1152 | - | AGAGGAAGTG | |
| ELK1 | 6.64747 | 0.833150710541 | 1281 | 1290 | + | GGACCAGAAG | |
| ELK1 | 6.60984 | 0.831900229436 | 252 | 261 | + | CAGAAGGAAG | |
| ELK1 | 6.42517 | 0.825763340694 | 666 | 675 | - | GTGAAGGAAG | |
